# ROS-triggered hydrophilicity switching synergizes with pH-responsive nanocarriers for therapy of diabetic wound

**DOI:** 10.1093/rb/rbaf098

**Published:** 2025-10-25

**Authors:** Bin Yin, Yueying Fan, Jinfu Li, Cheng Li, Shiqiang Jiang, Xiangyang Li, Chao Yan, Jiaxin Jiang, Peng Wang, Chiyu Jia

**Affiliations:** Center of Burn & Plastic and Wound Healing Surgery, The First Affiliated Hospital of University of South China, Hengyang Medical School, University of South China, Hengyang, Hunan 421001, China; Center of Burn & Plastic and Wound Healing Surgery, The First Affiliated Hospital of University of South China, Hengyang Medical School, University of South China, Hengyang, Hunan 421001, China; Center of Burn & Plastic and Wound Healing Surgery, The First Affiliated Hospital of University of South China, Hengyang Medical School, University of South China, Hengyang, Hunan 421001, China; Center of Burn & Plastic and Wound Healing Surgery, The First Affiliated Hospital of University of South China, Hengyang Medical School, University of South China, Hengyang, Hunan 421001, China; Center of Burn & Plastic and Wound Healing Surgery, The First Affiliated Hospital of University of South China, Hengyang Medical School, University of South China, Hengyang, Hunan 421001, China; Center of Burn & Plastic and Wound Healing Surgery, The First Affiliated Hospital of University of South China, Hengyang Medical School, University of South China, Hengyang, Hunan 421001, China; Center of Burn & Plastic and Wound Healing Surgery, The First Affiliated Hospital of University of South China, Hengyang Medical School, University of South China, Hengyang, Hunan 421001, China; Guangdong Huayi Biomedical Science and Technology Center, Guangzhou, Guangdong 511450, China; Center of Burn & Plastic and Wound Healing Surgery, The First Affiliated Hospital of University of South China, Hengyang Medical School, University of South China, Hengyang, Hunan 421001, China; Center of Burn & Plastic and Wound Healing Surgery, The First Affiliated Hospital of University of South China, Hengyang Medical School, University of South China, Hengyang, Hunan 421001, China

**Keywords:** ROS/pH dual-responsive, hydrophilicity switching, curcumin, anti-inflammatory, diabetic chronic wounds

## Abstract

Chronic diabetic wounds are notoriously difficult to heal due to the self-perpetuating cycle of persistent inflammation and oxidative stress, while current therapies are limited by single-action mechanisms and inefficient drug delivery. This study developed a reactive oxygen species (ROS)/pH dual-responsive hydrophilicity switching intelligent hydrogel (GC-HA@ZIF-8@Cur) by integrating a zeolitic imidazolate framework-8 (ZIF-8) with a dynamically crosslinked hydrogel for synergistic therapy. The system employs inflammation-targeting hyaluronic acid (HA)-modified ZIF-8 nanoparticles (HA@ZIF-8@Cur) to encapsulate curcumin (Cur), which are embedded into a ROS-responsive hydrogel matrix formed by ultraviolet-initiated polymerization of methacrylated gelatin and lipoic acid-grafted chitosan. In the ROS microenvironment of diabetic wounds, oxidation of thioether bonds in the hydrogel to sulfoxide bonds enhanced the hydrophilicity, while acidic conditions induced pH-responsive dissociation of ZIF-8 to cascade-release Cur and Zn^2+^. Experiments demonstrated that GC-HA@ZIF-8@Cur hydrogel reshapes the immune microenvironment by downregulating pro-inflammatory factors (interleukin [IL]-6, tumor necrosis factor [TNF]-α), polarizing macrophages toward the M2 phenotype, and upregulating IL-10, eliminating vascular generation disorders. Additionally, Zn^2+^ promotes vascular endothelial growth factor (VEGF) expression, accelerating angiogenesis. This dual-responsive system achieves spatiotemporally precise drug release, concurrently addressing inflammation, oxidative stress, and vascular regeneration barriers, significantly improving diabetic wound healing efficiency (96.372 ± 0.779% wound closure at day 14). It provides a novel multi-targeted co-delivery strategy for chronic wound therapy.

## Graphical Abstract

**Figure:**
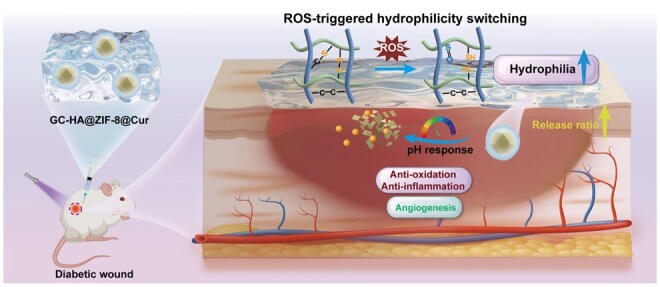

## Introduction

Chronic diabetic wounds represent one of the most devastating complications of diabetes [1]. An estimated 150 million diabetes sufferers worldwide are susceptible to chronic wound formation, where 15–25% of cases develop into lasting ulcers, causing in excess of a million amputations per annum [2, 3]. The core pathological mechanism lies in the synergistic interplay between persistent inflammation and excessive oxidative stress within the wound microenvironment [4]. During inflammatory responses, macrophages polarize toward the pro-inflammatory M1 phenotype, continuously releasing mediators, while significantly suppressing the expression of anti-inflammatory cytokines [5–7]. This process further exacerbates the inflammatory state and leads to excessive degradation of the extracellular matrix [8, 9]. Concurrently, reactive oxygen species (ROS) overproduction directly damages mitochondrial function, compromising endogenous antioxidant defenses [10, 11]. This leads to cell and tissue damage, which hinders angiogenesis and tissue repair [12–16]. Critically, ROS activates the NLRP3 inflammasome, promoting interleukin (IL)-1β maturation and amplifying inflammatory signaling, thereby establishing a self-reinforcing “inflammation-oxidative stress-tissue damage” loop [17]. This pathological cascade traps wounds in a prolonged inflammatory phase, with healing times often exceeding 12 weeks [18], necessitating targeted interventions to disrupt this vicious cycle.

Current therapeutic strategies for diabetic wounds commonly employ glucocorticoids (such as dexamethasone), non-steroidal anti-inflammatory drugs (such as indomethacin), and antioxidants (such as N-acetylcysteine) to mitigate inflammation and oxidative stress. However, these significant pharmacological drawbacks (such as inhibition of collagen synthesis, delay in re-epithelialization, short half-life, and the need for frequent administration) limit their capacity to address the multifactorial pathophysiology of diabetic wound healing [19–21]. Curcumin (Cur), a natural polyphenol with anti-inflammatory, antioxidant, and antimicrobial properties [22], has garnered significant attention due to its multitargeted effects and low toxicity. Cur inhibits NF-κB signaling to reduce pro-inflammatory cytokines and scavenges ROS to alleviate oxidative stress [23]. Although Cur demonstrates multifaceted therapeutic benefits in diabetic wound healing, its inherent hydrophobicity and suboptimal bioavailability remain critical challenges.

Recent advances in nanotechnology have addressed these limitations through innovative delivery systems. Metal–organic frameworks are crystalline porous materials composed of metal nodes linked by organic ligands. Their exceptional surface area, adjustable porosity, and modifiable surfaces make them attractive for drug delivery applications. Among them, zeolitic imidazolate framework-8 (ZIF-8) has attracted particular attention due to its unique physicochemical properties. Notably, ZIF-8 not only exhibits excellent biocompatibility and pH-responsive drug release characteristics, but its intrinsic Zn^2+^ release can also synergistically exert antibacterial effects and promote angiogenesis, making it an ideal platform for constructing intelligent wound therapy systems [24, 25]. For instance, Zhang et al. developed a thermosensitive injectable hydrogel co-loaded with ZIF-8 and quercetin, which demonstrated lubrication and microenvironment modulation capabilities, effectively improving osteoarthritis treatment [26]. He et al. further engineered a cerium dioxide-coated ZIF-8 composite incorporating the Rho-associated protein kinase inhibitor Y-27632, which achieved coordinated suppression of inflammation and tissue regeneration by regulating the dynamic balance between macrophage polarization and angiogenesis [27]. However, current delivery systems, largely restricted to single-stimulus-responsive mechanisms (e.g. pH- or enzyme-triggered release), are inadequate to address the complex, dynamic, and multi-factorial pathological microenvironment characteristic of chronic wounds, where concurrent stimuli like ROS bursts, MMP overexpression, and fluctuating pH gradients coexist [28, 29]. This mismatch between drug release kinetics and the temporal requirements of tissue repair significantly compromises the long-term efficacy and precision of treatment. Addressing this critical scientific challenge necessitates the development of smart delivery systems capable of multi-stimuli responsiveness and programmable drug release.

**Figure 1.**
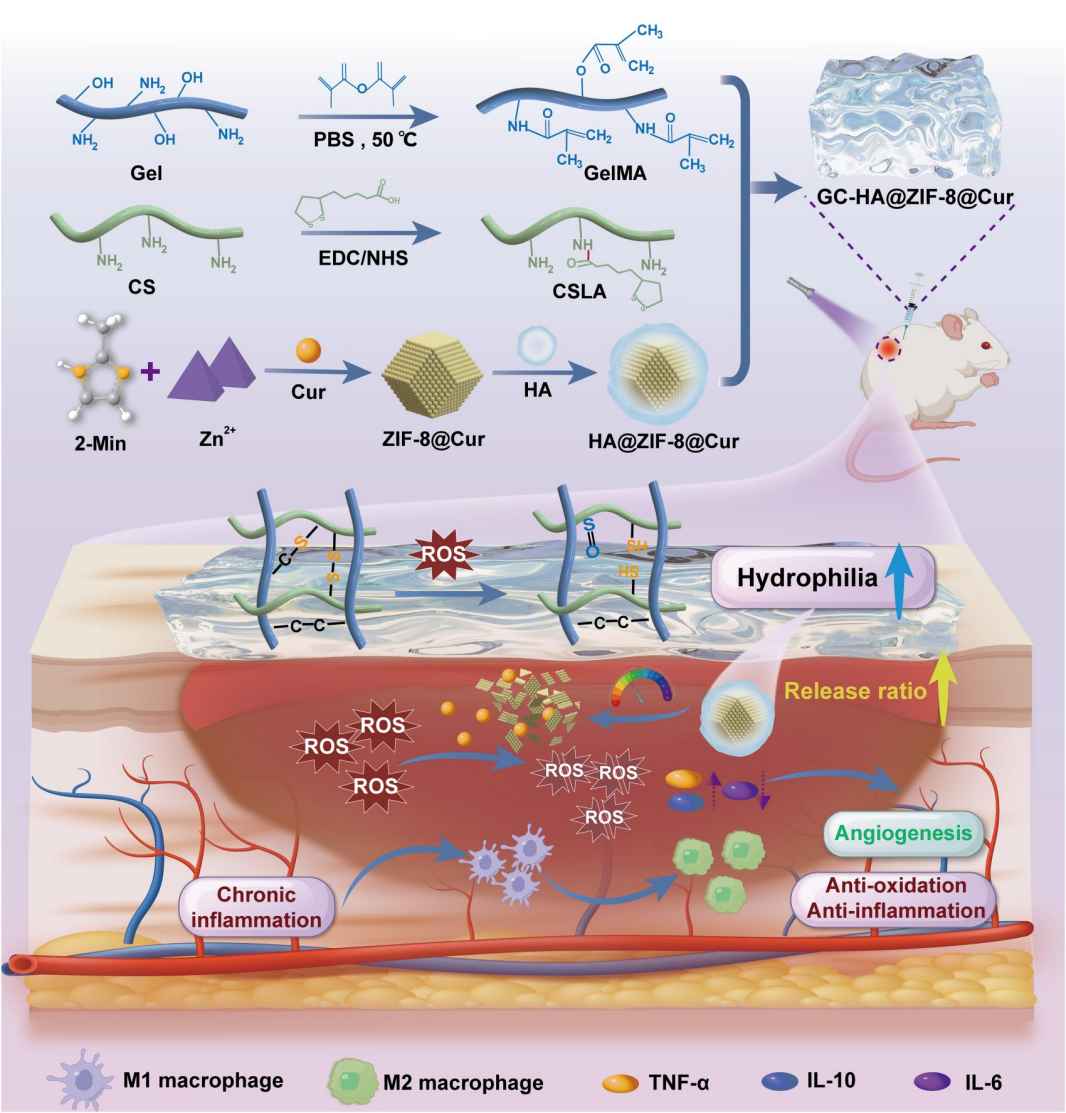
Schematic illustration of ROS-triggered hydrophilic switching synergized with pH-responsive nano system and its therapeutic mechanism for diabetic wound healing.

To address the aforementioned technical challenges, this study innovatively developed a ROS/pH dual-responsive intelligent hydrogel system (GC-HA@ZIF-8@Cur) for diabetic chronic wound therapy (Figure 1). The system features a functional core composed of ZIF-8 loaded *in situ* with Cur, which is further modified with hyaluronic acid (HA) to construct HA@ZIF-8@Cur nanoparticles. Building upon this foundation, a three-dimensional network hydrogel carrier with ROS-responsive properties was fabricated via ultraviolet (UV)-initiated crosslinking polymerization of methacrylated gelatin (GelMA) [30] and lipoic acid-grafted chitosan (CSLA) [31]. This intelligent release system enables spatiotemporally controlled drug delivery through a dual mechanism: The disulfide bonds in CSLA undergo specific cleavage to generate thiol groups, which subsequently form thioether bonds with double bonds in GelMA. In the high-ROS microenvironment of wounds, as the thioether bonds are progressively oxidized into hydrophilic sulfoxide structures, rapid release of HA@ZIF-8@Cur nanoparticles is achieved. Simultaneously, the nanoparticles themselves dissociate under the acidic pH stimulus of the wound, further releasing Cur in a cascade-like manner. The dual-responsive system not only remodels the immune microenvironment by polarizing macrophages from the M1 to M2 phenotype but also effectively scavenges excess ROS to alleviate oxidative stress while activating the expression of key angiogenic factors such as vascular endothelial growth factor (VEGF). This smart drug delivery strategy, which dynamically responds to pathological wound conditions, achieves synergistic effects combining anti-inflammatory, antioxidant, and pro-angiogenic properties, providing an innovative solution for establishing a stable microenvironment conducive to tissue regeneration. The system demonstrates significant potential for clinical applications in diabetic chronic wound therapy.

## Materials and methods

### Materials

Gelatin (Gel), Curcumin (Cur, > 98%), Zn (NO_3_)_2_·6H_2_O, N, N-dimethylformamide (DMF), methanol (MeOH), hyaluronic acid (HA, *Mw *= 200–400 kDa), Rhodamine B, Chitosan (CS), methacrylic anhydride (MA), ascorbic acid, hydrogen peroxide (H_2_O_2_), lipoic acid (LA), were purchased from Shanghai Macklin Biochemical Co., Ltd (Shanghai, China). Standard bacterial strains (*Escherichia coli* [*E. coli*], ATCC 8739, and *Staphylococcus aureus* [*S. aureus*], ATCC 6538) were obtained from the China General Microbiological Culture Collection. L-929 Mouse Fibroblast Cell Line (L929), Human Umbilical Vein Endothelial Cells (HUVEC), and Mouse Mononuclear Macrophage Cells (RAW264.7 macrophages) were sourced from Guangzhou Anbang Bioscience. All chemicals were analytical grade.

### The synthesis and characterization of HA@ZIF-8@Cur

ZIF-8@Cur was prepared via nano-precipitation. Solution A contained Zn(NO_3_)_2_·6H_2_O (2.14 g) in DMF/MeOH (4:1 v/v), while solution B contained 2-methylimidazole (2.32 g) and Cur (0.5 g) in the same solvent system. After dropwise mixing and 2 h stirring, the product was centrifuged (10 000 rpm, 10 min), methanol-washed, and vacuum-dried. ZIF-8 was similarly synthesized without Cur.

HA@ZIF-8@Cur was synthesized following established methods [32]. Briefly, HA (4 mg) and ZIF-8@Cur (8 mg) were dispersed in ultrapure water (4 mL) and stirred for 3 h at room temperature (RT). The reaction was terminated with ethanol (0.4 mL), followed by centrifugation and lyophilization. Rhodamine B-labeled nanoparticles were similarly prepared for cellular uptake studies.

The nanoparticles were systematically characterized using complementary analytical techniques. Morphological examination and elemental distribution analysis were performed by transmission electron microscopy (TEM, JEOL JEM-2100F), while energy dispersive X-ray spectroscopy provided detailed information about elemental composition and chemical states. The surface charge properties were evaluated through dynamic light scattering measurements (DLS, Nano-Brook 90PlusZata) of aqueous nanoparticle suspensions. The Brunauer–Emmett–Teller (BET) method was employed to measure surface area and pore size, and optical properties were assessed using UV-Vis spectrophotometry (PerkinElmer Lambda 750) to identify characteristic absorption peaks.

The encapsulation efficiency (EE) and drug loading capacity (DLC) of Cur loaded into ZIF-8 were determined through UV-Vis spectrophotometric analysis at 422 nm. A standard curve of Cur was first established, and the absorbance of both the initial Cur solution and the supernatant after ZIF-8@Cur preparation was measured to calculate their respective concentrations using the standard curve. The EE was calculated using the (1) formula:


(1) $$\mathrm{EE}\;(\%)=(\frac{C_{I}-C_{S}}{C_{I}})\times 100\%,$$

where *C*_I_ represents the initial concentration of Cur and *C*_S_ denotes the concentration of Cur in the supernatant after encapsulation.

For DLC measurement, 1 mg/mL of ZIF-8@Cur nanoparticles was dispersed in an ethanol/HCl (1:1) solution and stirred at RT for 24 h to completely release the loaded Cur, followed by centrifugation to separate the nanoparticles. The absorbance of the resulting supernatant was measured and converted to Cur concentration. The DLC was calculated using the (2) formula:


(2) $$\mathrm{DLC}\;(\mathrm{mg}/g)=\frac{m_{\mathrm{cur}}}{m_{T}},$$

where *m*_Cur_ is the mass of loaded Cur and *m*_T_ is the total mass of ZIF-8@Cur nanoparticles.

Determination of Zn^2+^ content in HA@ZIF-8@Cur using Inductively Coupled Plasma Optical Emission Spectroscopy (ICP-OES, Agilent-720). Approximately 5 mg of HA@ZIF-8@Cur was weighed and transferred into a polytetrafluoroethylene digestion vessel. Subsequently, 5 ml of concentrated nitric acid (HNO_3_, 65%) was added, and the vessel was sealed for microwave-assisted digestion. After digestion, the sample was cooled to RT, and the resulting solution was diluted to 50 ml with ultrapure water in a volumetric flask. The Zn^2+^ content in HA@ZIF-8@Cur was then quantitatively analyzed.

### The synthesis and characterization of GelMA and CSLA

GelMA was synthesized by dissolving gelatin (10 g) in 100 ml of bicarbonate buffer at 50°C. Methacrylic anhydride (0.375 mL) was added to the solution under alkaline conditions (pH 9, adjusted with 1 M NaOH). Following 3 h of reaction, the mixture was neutralized with 1 M HCl, then purified through dialysis (MWCO 3.5 kDa, 5 days) and lyophilized.

CS (1.0 g) was dissolved in 90 ml of 2% acetic acid. The reaction was initiated by adding ascorbic acid (0.1 g) and H_2_O_2_ (1.3 mL, 7.7 wt%) as catalysts with 30 min stirring. LA (0.25 g in ethanol) was then introduced and reacted for 24 h at RT. After pH neutralization with NaOH, the product was dialyzed (MWCO 3.5 kDa, 3 days) and lyophilized to yield CSLA [33]. GelMA and CSLA structures were verified by proton nuclear magnetic resonance spectroscopy (^1^H NMR, Bruker 400 MHz) and Fourier transform infrared spectroscopy (FTIR, Thermo Fisher iS5).

### The synthesis and characterization of GC- HA@ZIF-8@Cur

The composite hydrogel was prepared by homogenizing GelMA (15 wt%), CSLA (2 wt%), and HA@ZIF-8@Cur (0.2 wt%), followed by 3-min equilibration and 5-min UV crosslinking to form GC-HA@ZIF-8@Cur. The morphology of the freeze-dried hydrogel was characterized by scanning electron microscopy (SEM, CIQTE SEM5000X).

Mechanical properties were evaluated using a Shimadzu universal testing machine. For tensile testing, dumbbell-shaped hydrogels (30 × 4 × 2 mm) were stretched at 10 mm/min to determine tensile strength and breaking strain. Cylindrical samples (*Φ*10 × 10 mm) were compressed at 1 mm/min to measure compressive strength and failure strain.

Swelling behavior was assessed by incubating hydrogels in PBS at 37°C (*n *= 3). Periodic weight measurements were taken to calculate the swelling ratio using the following formula:


(3) $$\text{Swelling}\;\text{ratio}\;(\%)=\frac{W_{t}-W_{0}}{W_{0}}\times 100\%,$$

where *W*_0_ is the initial mass and *W*_t_ represents the mass at measurement time points.

Hydrogel hydrophilicity was evaluated using an optical contact angle analyzer. Measurements were performed by depositing 3 μl water droplets and imaging after 20 s stabilization, with triplicate testing for statistical reliability.

Viscoelasticity test: The precursor solution of the photosensitive pre-polymer hydrogel was subjected to a time scan under 10 mW/m^2^ ultraviolet irradiation using a rheometer (Anton Paar-MCR 302e), with a constant strain rate of 1.0% and an angular frequency of 10.0 rad/s.

### Degradation performance

Immerse GC-HA@ZIF-8@Cur in 5 mL of PBS at 37°C. Replace the PBS every 3 days. After different degradation days, freeze-dry the samples and measure their weight. Record the mass and calculate the mass remaining ratio. In addition, the morphology of the samples degraded for 7 and 14 days was characterized by SEM.

### *In vitro* drug release experiment

Drug release profiles were assessed by encapsulating hydrogels in dialysis bags (MWCO 1 kDa) and incubating in PBS (pH 7.4 or 6.4) with/without 1 mM H_2_O_2_ at 37°C under agitation. Release medium aliquots were periodically collected and replaced, with Cur quantification performed via UV-Vis spectrophotometer at 280 nm.

### *In vitro* antioxidant properties of hydrogels

The antioxidant properties were assessed by 1,1-diphenyl-2-picrylhydrazine (DPPH) and 2, 2′-azino-bis (3-ethylbenzothiazoline-6-sulfonic acid) (ABTS) radical scavenging assay. GC, HA@ZIF-8, Cur, HA@ZIF-8@Cur, and GC-HA@ZIF-8@Cur (200 μL) were added to the 1.8 mL ethanol solution containing DPPH or ABTS (1.5 mM), and the mixtures were placed in a dark place for 30 min, and vitamin C was used as the positive control group among them. The supernatant was then removed and scanned using a UV-Vis spectrophotometer at 517 and 734 nm, respectively.


(4) $$\text{DPPH}\;\text{scavenging}\;(\%)=\frac{A_{N}-A_{S}}{A_{N}-A_{P}}\times 100\%$$

(5) $$\text{ABTS}\;\text{scavenging}\;(\%)=\frac{A_{N}-A_{S}}{A_{N}-A_{P}}\times 100\%,$$

where *A*_N_, *A*_S_, and *A*_P_ correspond to the absorbance readings of the negative control, test sample, and positive control, respectively.

### *In vitro* antibacterial experiment

Antibacterial activity was evaluated by co-culturing *S. aureus* and *E. coli* with various hydrogel samples in Luria–Bertani (LB) medium. Bacterial growth was monitored by measuring OD620 at different time intervals, while colony counting was performed after 8-h co-culture on LB agar plates. The formula for calculating the antibacterial ratio is:


(6) $$\text{Antibacterial}\;\text{ratio}\;(\%)=\frac{N_{\mathrm{blank}}-N_{\mathrm{hydrogel}}}{N_{\mathrm{blank}}}\times 100\%,$$

where *N*_blank_ and *N*_hydrogel_ represent the number of bacterial colonies in the blank group and the materials group, respectively.

### Hemolysis assay

Hemocompatibility was assessed through *in vitro* hemolysis testing. After incubating hydrogels with erythrocytes (37°C, 3 h) and centrifugation (4000 rpm, 10 min), the supernatant was diluted with PBS (100 μL + 2.5 mL). Hemolysis rates were calculated from OD540 measurements using the following formulas:


(7) $$\text{Hemolysis}\;\text{ratio}\;(\%)=\frac{{OD}_{\mathrm{Sample}}-{OD}_{\mathrm{Negative}}}{{OD}_{\mathrm{Positive}}-{OD}_{\mathrm{Negative}}\;}\times 100\%$$

*OD*_Sample_, *OD*_Negative_, and *OD*_Positive_ represent the OD value of samples, PBS, and deionized water, respectively. All experiments were examined 3 times.

### Live/dead cell double staining assay and CCK8 assay

Hydrogel biosafety was assessed using Cell Counting Kit-8 (CCK-8) and live/dead staining. L929 cells (1 × 10^4^/well) were cultured with hydrogel samples for 24/48 h, followed by CCK-8 treatment (100 μL) and OD450 measurement. Parallel live/dead staining (FITC/PI) in six-well plates was imaged using fluorescence microscopy (Olympus CKX53).

### Cell uptake assay

HUVEC uptake of HA@ZIF-8@Cur was assessed by seeding cells in 12-well plates (1 × 10^5^/well) for 24 h, then treating with rhodamine B-loaded nanoparticles for 12 h. After DAPI (nuclei) and FITC-phalloidin (cytoskeleton) staining, samples were imaged using a fluorescence microscope (Olympus CKX53), with PBS as a control.

### Evaluation of the ability to clear ROS and induce macrophage polarization

Intracellular ROS scavenging was evaluated using DCFH-DA. RAW264.7 cells in 24-well plates were treated with hydrogels in 0.1 mM H_2_O_2_ for 4 h (PBS as negative control), then stained with DCFH-DA (20 min) and imaged by fluorescence microscopy after PBS washing.

Macrophage polarization was assessed to evaluate anti-inflammatory activity. LPS-stimulated RAW264.7 cells (1 μg/mL, 4 h) were treated with hydrogels, fixed (15 min), and immunostained with iNOS/CD206 antibodies (Abcam) overnight at 4°C. After secondary antibody incubation (30 min, dark), samples were imaged by confocal microscopy, with PBS-treated cells as a control.

### Transwell assay

HUVEC migration was assessed using transwell assays. Hydrogel samples in the lower chamber (DMEM/F12) attracted cells seeded in the upper chamber (200 μL, 12 h at 37°C). Migrated cells were fixed (4% PFA), crystal violet stained.

### *In vitro* scratch assay

HUVECs (4 × 10^4^ cells/well) were grown to 90–95% confluence in 24-well plates before creating uniform wounds with pipette tips. Following 24-h treatment with 500-μL material extracts, migration distances were quantified using inverted microscopy.

### Tube formation assay

To assess material angiogenesis, a tube formation assay was performed. Briefly, 100 μL of chilled matrix hydrogel was added to 24-well plates and solidified at 37°C for 30 min. HUVECs (3 × 10^4^ cells/well) were then cultured with GC, HA@ZIF-8@Cur, and GC-HA@ZIF-8@Cur for 12 h. Tube structures were examined by fluorescence microscopy and quantified using ImageJ.

### *In vivo* diabetic wound healing of the hydrogels

Our animal experiments were performed in compliance with the National Research Council’s Guide for Laboratory Animal Care and Use, approved by the Ethics Committee for Laboratory Animals of the University of South China (approval number: 11000302-1). To induce diabetes in the experimental rats, 32 male Sprague–Dawley (SD) rats received an intraperitoneal injection of streptozotocin (STZ, 65 mg/kg) 5 days prior to wound induction. Diabetic status was confirmed by sustained hyperglycemia (>300 mg/dL) through repeated blood glucose measurements. Subsequently, the rats were anesthetized using sodium pentobarbital (50 mg/kg), followed by dorsal hair removal and the creation of 15-mm full-thickness circular excisional wounds. The rats were then divided into four experimental groups: control, GC, HA@ZIF-8@Cur, and GC-HA@ZIF-8@Cur. Wound closure was assessed at 1, 3, 5, 9, and 14 days post-treatment, with healing rates quantified through ImageJ analysis. Wound healing ratio calculation:


(8) $$\text{Wound}\;\text{healing}\;\text{ratio}\;(\%)=\frac{S_{0}-S_{t}}{S_{t}}\times 100\%,$$

where *S*_0_ denotes the original wound size, while *S*_t_ indicates the wound area at measurement time.

On days 7 and 14, wound tissues were harvested from euthanized SD rats and sectioned (5 μm) for histological analysis. Masson’s trichrome and hematoxylin-eosin (H&E) staining evaluated tissue regeneration, while IL-6, IL-10, and tumor necrosis factor (TNF)-α immunohistochemistry assessed inflammation.

On day 7 post-injury, angiogenesis was assessed by immunofluorescence staining of CD31 and α-SMA [34] and VEGF immunohistochemistry [35]. Tissue sections underwent sequential incubation with primary antibodies at 4°C overnight, followed by secondary antibodies for 30 minutes, and counterstained with DAPI. Images were acquired using CLSM.

### Statistical analysis

Statistical analysis was conducted using Origin with triplicate experiments (mean ± SD). Data were evaluated by one-way ANOVA with Tukey’s test or unpaired *t*-test. Significance levels: *P < 0.05, **P < 0.01, ***P < 0.001.

## Results

### Preparation and characterization of HA@ZIF-8@Cur

**Figure 2.**
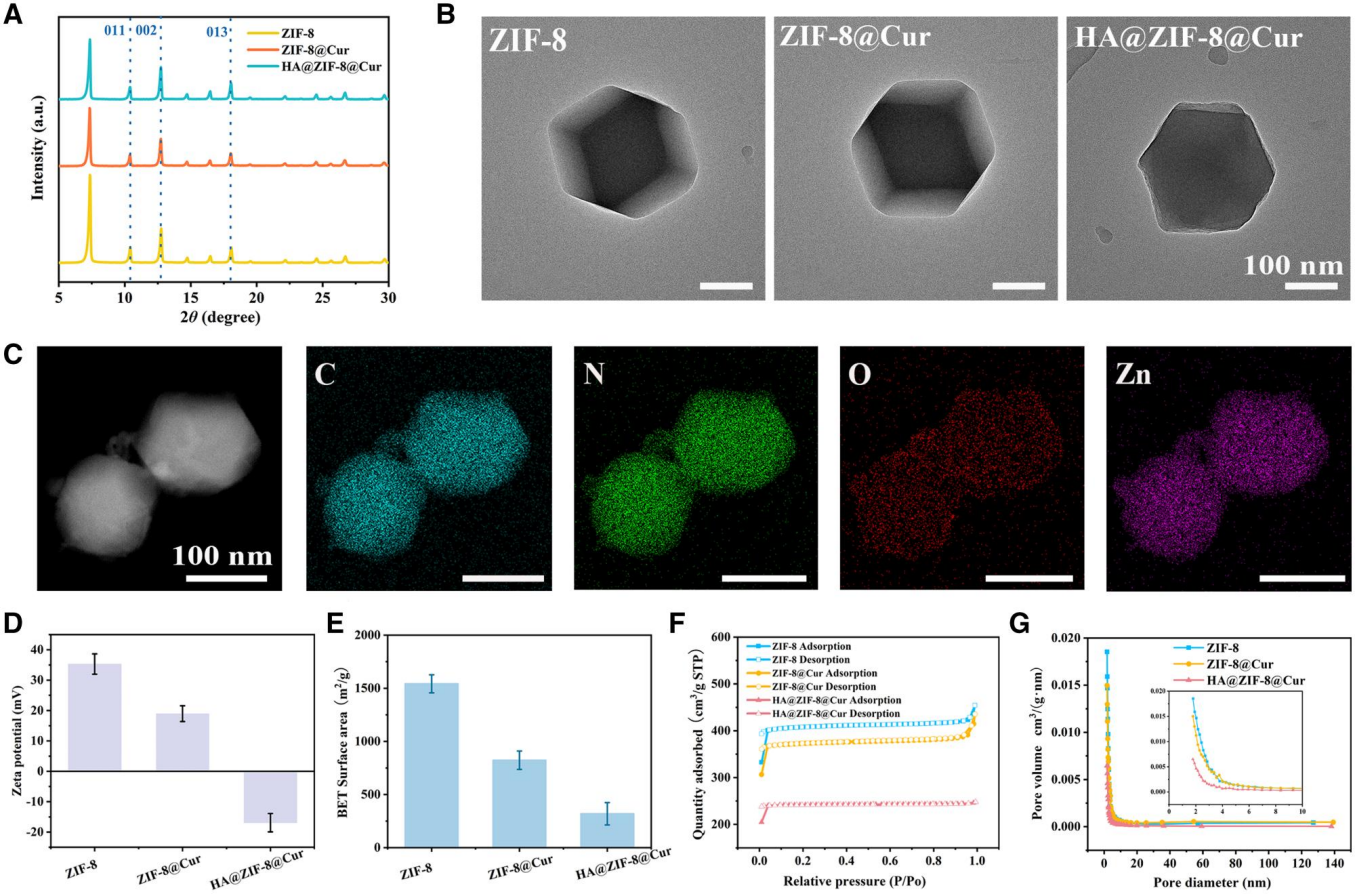
Structural characterization of HA@ZIF-8@Cur nanocomposite. (**A**) Comparative XRD of ZIF-8, ZIF-8@Cur, and HA@ZIF-8@Cur. (**B**) TEM illustrating morphological features of the three materials. (**C**) Elemental mapping of HA@ZIF-8@Cur. (**D**) Surface charge measurements via zeta potential analysis. (**E**) BET surface area quantification. (**F** and **G**) Nitrogen physisorption isotherms and corresponding pore size distribution analyses. Data is expressed as SD ± average.

The nano-co-precipitation method effectively produced HA@ZIF-8@Cur nanoparticles. A standard curve of Cur was plotted using a UV-Vis spectrophotometer first (Supplementary Figure S1), and the EE and DLC of Cur loaded into ZIF-8 were calculated to be 12.32 ± 0.35% and 104.08 ± 3.82 mg/g, respectively. Concurrently, the content of Zn^2+^ in HA@ZIF-8@Cur nanoparticles was determined to be 228.29 mg/g by ICP-OES. XRD patterns showed characteristic ZIF-8 peaks at 7.3° (011), 10.3° (002), and 16.4° (013) (Figure 2A). These results confirm the intact crystalline framework of ZIF-8 after Cur encapsulation and HA surface functionalization. TEM imaging further demonstrated that the nanoparticles maintained a uniform hexagonal morphology with an average diameter of 200 nm (Figure 2B), indicating that neither Cur loading nor HA coating disrupted the structural integrity of ZIF-8. Notably, the HA encapsulation layer was distinctly visible in HA@ZIF-8@Cur. Furthermore, the elemental mapping revealed a uniform distribution of C, N, O, and Zn elements in HA@ZIF-8@Cur (Figure 2C). The zeta potential tests showed that the potentials of ZIF-8, ZIF-8@Cur, and HA@ZIF-8@Cur were +35.29 ± 3.35 mV, +18.97 ± 2.60 mV, and −16.91 ± 3.01 mV, respectively. This trend can be attributed to the introduction of Cur and the HA surface modification, which progressively alter the surface charge characteristics (Figure 2D). Additionally, BET analysis shows that the specific surface area of ZIF-8 is 1542.35 ± 84.676 m^2^/g, which decreases to 823.17 ± 86.184 m^2^/g after loading Cur and further decreases to 319.22 ± 105.295 m^2^/g after coating with HA (Figure 2E). Adsorption analysis (Figure 2F) shows that the adsorption capacity of ZIF-8 is approximately 550 cm^3^/g, which reduces to about 400 cm^3^/g after loading Cur and further reduces to about 200 cm^3^/g after coating with HA. Pore size analysis (Figure 2G) shows that the pore size distribution of ZIF-8 is mainly concentrated at 1.80–6.60 nm, while the porosity volume densities of ZIF-8@Cur and HA@ZIF-8@Cur are successively reduced, being 0.015 and 0.0064 cm^3^/(g·nm), respectively, indicating that the total pore volume of the material changes with the loading of Cur and HA. These observations collectively suggest that Cur incorporation partially occupies ZIF-8 pores, while HA coating induces additional pore blockage, corroborating the successful stepwise fabrication of HA@ZIF-8@Cur.

### Characterization, mechanical properties, drug release behavior, and degradation performance of hydrogels

**Figure 3.**
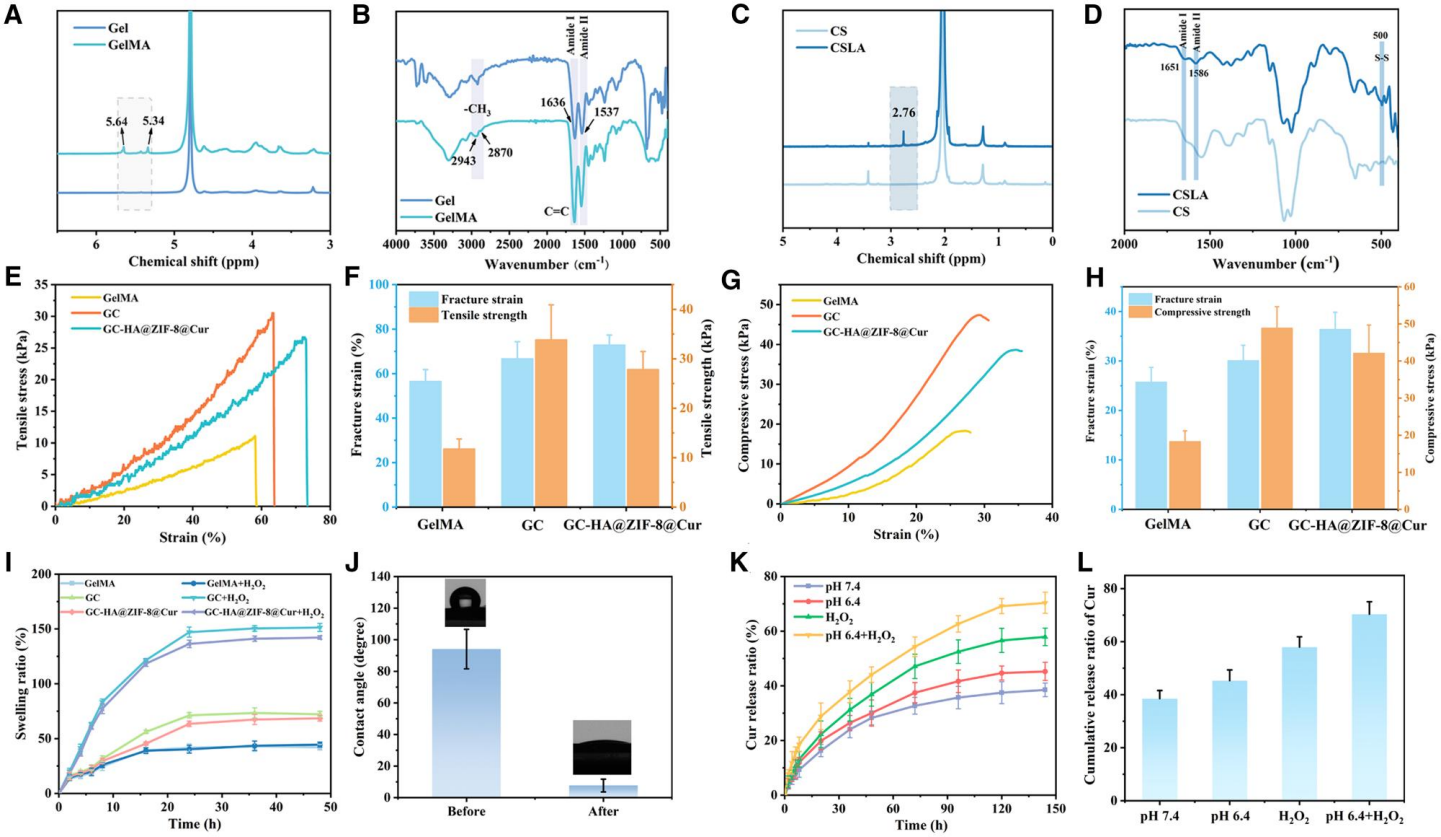
Synthetic characterization and mechanical properties of hydrogels. (**A**) ^1^H-NMR of gel and GelMA. (**B**) FTIR of gel and GelMA. (**C**) ^1^H-NMR of CS and CSLA. (**D**) FTIR of CS and CSLA. (**E**) Tensile stress-strain curves of hydrogels. (**F**) Tensile fracture strain and tensile strength of hydrogels. (**G**) Compression stress–strain curves of hydrogels. (**H**) Compression fracture strain and tensile strength of hydrogels. (**I**) Swelling curves of different hydrogels under different environments. (**J**) Water contact angle of GC-HA@ZIF-8@Cur before and after ROS treatment. (**K**) Drug release curves of GC-HA@ZIF-8@Cur in different environments. (**L**) Cumulative release of drugs from GC-HA@ZIF-8@Cur in different environments. (*n *= 3). Data is expressed as SD ± average.

In order to prepare a composite hydrogel, GelMA and CSLA need to be synthesized respectively. Firstly, the synthesis of GelMA was confirmed using ^1^H-NMR and FTIR spectroscopy. Figure 3A displays characteristic peaks at approximately 5.34 and 5.64 ppm, corresponding to the protons present on the methacryloyl group’s double bond [36]. Meanwhile, the FTIR spectroscopy results showed that both Gel and GelMA exhibited strong absorption peaks of Amide I (C=O) and Amide II (N–H) at 1636 and 1537 cm^−1,^ respectively. Due to the introduction of C=C, GelMA had a stronger characteristic peak at 1636 cm^−1^. Additionally, –CH_3_ was observed at 2943 and 2870 cm^−1^, confirming the successful preparation of GelMA (Figure 3B). Subsequently, according to the ^1^H-NMR spectra of CSLA (Figure 3C), the peaks observed at 2–3 ppm in the CSLA spectrum were assigned to the aromatic protons of LA, confirming the successful incorporation of LA. At the same time, the FT-IR spectra (Figure 3D) reveal new absorption bands at approximately 1647, 1586, and 500 cm^−1^, corresponding to the stretching vibration of the newly introduced C=O group, the bending vibrations of C–N/N–H, and S–S bonds, respectively. These findings further confirm the successful preparation of CSLA. Rheological tests recorded the curing process of GelMA, GC, and GC-HA@ZIF-8@Cur under UV light (Supplementary Figure S2). The crossover points of storage modulus (G′) and loss modulus (G″) indicated that the curing times of the three materials were very similar. After curing, GC exhibited a higher storage modulus than GelMA, whereas the introduction of ZIF-8@Cur nanoparticles led to physical crosslinking, which hindered some chemical crosslinking, resulting in a relatively lower storage modulus for GC-HA@ZIF-8@Cur. Subsequent SEM morphological characterization of the freeze-dried hydrogels (Supplementary Figure S3) revealed that GC, due to its highly dense crosslinked network, displayed a rich and tightly packed porous structure. In contrast, GC-HA@ZIF-8@Cur showed larger pore sizes but a more disordered crosslinked network, which was consistent with the observed decrease in G′ in the rheological tests.

To evaluate the mechanical properties of hydrogels, the compression properties of CSLA hydrogels of different concentrations were compared (Supplementary Figure S4) first. As the concentration of CSLA increased, the mechanical properties of GC hydrogels improved. Tensile and compression testing were conducted on GelMA, GC, and GC-HA@ZIF-8@Cur. Tensile testing results show that the fracture strains of GelMA, GC, and GC-HA@ZIF-8@Cur are 56.403 ± 5.402%, 66.573 ± 7.765%, and 72.743 ± 4.611%, respectively, while their tensile strengths are 11.727 ± 2.069 kPa, 33.800 ± 7.113 kPa, and 27.817 ± 3.653 kPa, respectively (Figure 3E and F). Compression testing results indicate that the fracture strains of GelMA, GC, and GC-HA@ZIF-8@Cur were 25.727 ± 2.973%, 30.073 ± 3.114%, and 36.400 ± 3.454%, respectively, and their compressive strengths were 18.287 ± 2.885 kPa, 48.857 ± 5.766 kPa, and 42.077 ± 7.589 kPa, respectively (Figure 3G and H). Intuitive compression images were shown in Figure S5. These results demonstrate that the double-component UV polymerized hydrogel GC has significantly improved mechanical properties compared to the single-component hydrogel GelMA. The mechanical properties of the GC-HA@ZIF-8@Cur hydrogel have slightly decreased, and this is consistent with the trends observed in rheology and SEM. In addition, the swelling test showed that the hydrogel reached swelling equilibrium after 24 h. Notably, the swelling ratio of the GC group and the GC-HA@ZIF-8@Cur group was comparable, suggesting that the incorporation of nanomaterials did not significantly affect the hydrogel’s swelling performance. Subsequently, upon H_2_O_2_ stimulation, the swelling ratios of both GC and GC-HA@ZIF-8@Cur hydrogels increased significantly by 2.0-fold compared to those in a normal environment (Figure 3I). This finding could be attributed to the formation of hydrophilic sulfoxide bonds triggered by ROS, which strengthened intermolecular hydrophilic interactions to promote water uptake. To investigate changes in hydrophilicity, we measured the contact angle before and after H_2_O_2_ treatment (Figure 3J). The experimental results showed that after H_2_O_2_ treatment, the contact angle of GC-HA@ZIF-8@Cur decreased substantially from 94.098 ± 12.480° (hydrophobic) to 7.730 ± 4.044° (hydrophilic). This phenomenon can be attributed to the oxidation of thioether bonds within the hydrogel to hydrophilic sulfoxide bonds induced by ROS. The improvement in hydrophilicity will enhance the biocompatibility and drug release performance of the GC-HA@ZIF-8@Cur hydrogel.

The drug release profiles of Cur from GC-HA@ZIF-8@Cur hydrogel were examined in various media. The findings indicated that the Cur release from GC-HA@ZIF-8@Cur was significantly higher in acidic conditions (pH 6.4) than in neutral conditions (pH 7.4), which can be attributed to the degradation of ZIF-8 in the acidic environment. In addition, in the environment containing H_2_O_2_, the Cur release was further increased. Under acidic and H_2_O_2_ conditions, the cumulative release ratio over a period of 80 h exceeded 60% (Figure 3K and L). This is attributed to ROS oxidizing the disulfide bonds in the hydrogel into hydrophilic sulfoxide bonds, thereby accelerating drug release. This mechanism highlights the hydrogel’s ability to release drugs efficiently in low-pH, high-ROS environments, supporting its potential application in diabetic wound therapy. Subsequently, kinetic fitting analysis was performed to elucidate the drug release mechanism of GC-HA@ZIF-8@Cur. The results showed that the Korsmeyer–Peppas model exhibited the highest determination coefficients (*R*^2^ > 0.97) across all conditions, outperforming zero-order, first-order, and Higuchi models. Additionally, the diffusion index values of the Korsmeyer–Peppas model ranged from 0.512–0.587, indicating that the drug release behavior of GC-HA@ZIF-8@Cur conformed to non-Fickian diffusion mechanism and was regulated by a combination of diffusion and swelling mechanisms in different environments. Moreover, the release ratio of Cur reached its maximum under acidic and H_2_O_2_ conditions (Supplementary Figure S6A–D). This microenvironment-responsive release characteristic can be ascribed to the synergistic effects of pH-triggered ZIF-8 dissociation and ROS-induced alterations in the hydrophilic-hydrophobic balance of the hydrogel, enabling controlled release of Cur. These results establish a robust theoretical foundation for GC-HA@ZIF-8@Cur to achieve responsive release within the diabetic wound microenvironment.

To ensure the degradation behavior of the wound dressing matches the tissue repair cycle, this study evaluated the degradation characteristics of GC-HA@ZIF-8@Cur. The degradation studies demonstrated that the GC-HA@ZIF-8@Cur hydrogel gradually lost its well-ordered structure (Supplementary Figure S7) when incubated in PBS at 37°C over time, while maintaining a mass retention rate of 63.78 ± 3.13% after 14 days (Supplementary Figure S8). Additionally, the cumulative Zn^2+^ release ratio from GC-HA@ZIF-8@Cur in the degradation medium was quantified as 23.77 ± 1.55% using ICP spectroscopy at day 14. These findings demonstrate that GC-HA@ZIF-8@Cur possesses controllable degradation properties that align well with the wound healing timeline while preventing premature functional failure due to excessive degradation.

### Antioxidant and antibacterial properties of hydrogels

**Figure 4.**
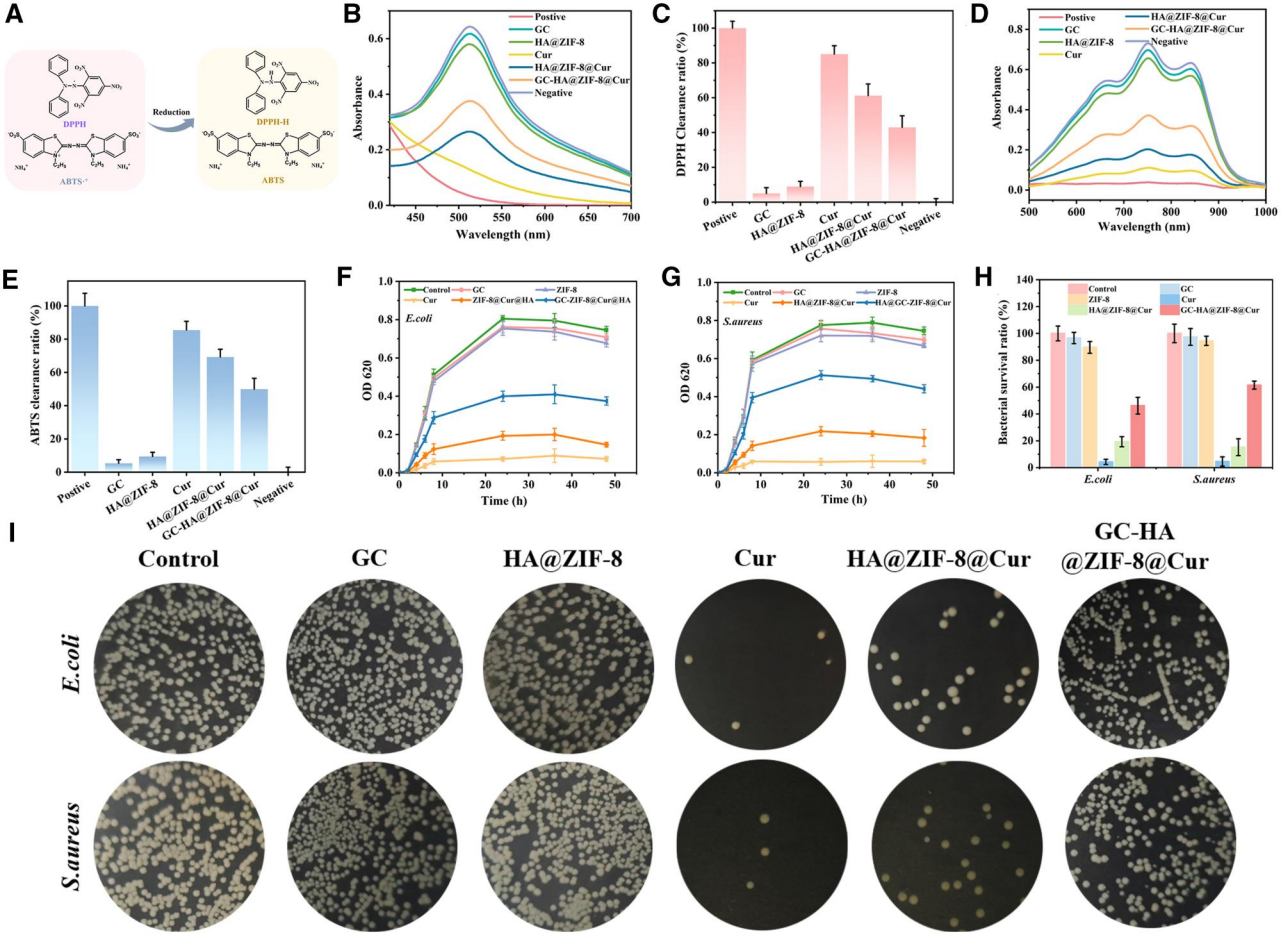
ROS Clearance and antibacterial properties of hydrogel. (**A**) Reduction mechanism diagram of DPPH and ABTS free radicals. (**B**) DPPH free radical scavenging curves of different materials. (**C**) DPPH free radical clearance of different materials. (**D**) ABTS radical scavenging curves of different materials. (**E**) ABTS free radical clearance of different materials. Bacterial growth kinetics analysis (**F**) *E. coli* and (**G**) *S. aureus* proliferation profiles during 48-hour material exposure. (**H**) Quantitative viability assessment showing bacterial survival percentages after 8-hour treatments. (**I**) Colony morphology documentation of both gram-negative (*E. coli*) and gram-positive (*S. aureus*) bacteria following 8-hour material incubation.

To assess the antioxidant properties of hydrogels, we evaluated their ROS scavenging capacity by measuring the DPPH and ABTS free radical scavenging efficiencies (Figure 4A). The experimental data revealed that Cur, HA@ZIF-8@Cur, and GC-HA@ZIF-8@Cur exhibited significant decreases in UV absorption at wavelengths of 517 and 734 nm, indicating their potent ROS scavenging capabilities. In contrast, GC and ZIF-8 showed minimal changes in absorption, suggesting relatively weaker ROS scavenging abilities. Notably, the absorption intensity of Cur was comparable to that of the positive control group, which indicates that it has an excellent ability to clear ROS (Figure 4B and D). This finding was further corroborated by the quantitative histogram of free radical clearance (Figure 4C and E). Collectively, these results indicate that GC-HA@ZIF-8@Cur possesses outstanding antioxidant properties, primarily attributable to the ROS scavenging activity of Cur.

Bacterial infection is a critical factor affecting wound healing [37]. Therefore, we used the turbidity method and the dilution plating method to detect the antibacterial ability of the hydrogel *in vitro*. As shown in Figure 4F and G, the results demonstrated that the Cur group exhibited the strongest antibacterial performance, while the GC and HA@ZIF-8 groups showed negligible activity. By comparing the HA@ZIF-8 and HA@ZIF-8@Cur groups, it was clearly determined that the main antibacterial performance was attributed to Cur. However, due to the encapsulation and sustained-release effect of the GC hydrogel on the HA@ZIF-8@Cur nanoparticles, the HA@ZIF-8@Cur group had a stronger antibacterial effect compared to the GC-HA@ZIF-8@Cur group. Meanwhile, the same results were obtained according to the image of bacterial colony and bacterial survival ratio (Figure 4H and I). These results indicate that the introduction of cur significantly improved the antibacterial activity of GC-HA@ZIF-8@Cur.

### Biocompatibility and angiogenesis analysis of hydrogels

**Figure 5.**
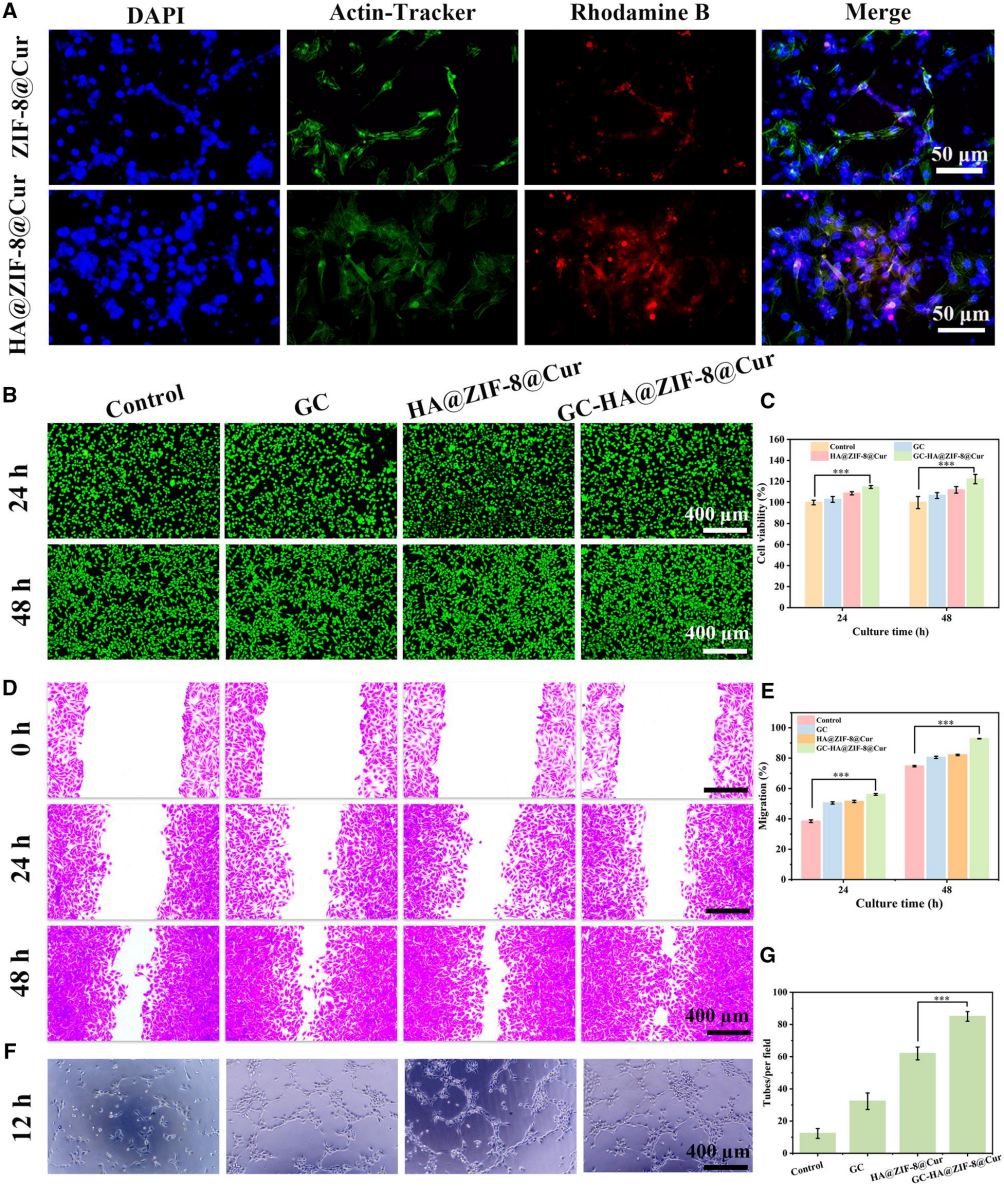
Cytocompatibility evaluation of hydrogel components. (**A**) Intracellular nanoparticle uptake visualized by confocal microscopy in HUVECs treated with ZIF-8@Cur and HA@ZIF-8@Cur. Viability of L929 fibroblasts as assessed (**B**) by live/dead staining and (**C**) by CCK-8 assay after 24 and 48 h of exposure to the material. (**D**) Endothelial cell migration patterns and (**E**) quantitative migration ratios after 24-48 h treatments. (**F**) Angiogenic potential demonstrated by capillary-like structure formation and (**G**) quantitative tube formation analysis after 12 h incubation. (*n *= 3). Data is expressed as SD ± average. *** P < 0.001.

Nanodrugs can be effectively internalized by cells, thereby significantly enhancing their anti-inflammatory and antioxidant effects while accelerating wound healing. The cellular uptake ability of the materials was evaluated using HUVECs through fluorescence imaging analysis. The results showed that the HA@ZIF-8@Cur group exhibited significantly enhanced red fluorescence signals within the cells, indicating higher cellular uptake efficiency (Figure 5A). This phenomenon might be attributed to the excellent biocompatibility of HA. Optimal biocompatibility, particularly concerning cytotoxicity and hemolytic properties, serves as a fundamental requirement for hydrogel applications [38]. Blood compatibility was assessed through hemolysis testing utilizing freshly isolated murine erythrocytes. The results showed that there was no hemolysis in the red blood cell lysate after GC-HA@ZIF-8@Cur treatment, and the hemolysis ratio was <0.3% (Supplementary Figure S9). Biocompatibility evaluation was conducted through dual approaches: live/dead fluorescence staining and CCK-8 metabolic assay in L929 fibroblast cultures. The image showed significantly enhanced cellular proliferation in GC-HA@ZIF-8@Cur-treated samples versus controls at 48 h (Figure 5B). The CCK-8 results confirmed exceptional cell viability (>95%) (Figure 5C), with GC-HA@ZIF-8@Cur exhibiting the most favorable proliferation outcomes. Notably, GC-HA@ZIF-8@Cur exhibited significant cell proliferation, suggesting that the hydrogel promotes cell growth. This phenomenon may be attributed to Zn^2+^ acting as a cofactor for key enzymes and transcription factors, thereby regulating the expression of numerous genes involved in cell cycle control, proliferation, and survival [33]. Additionally, zinc ions can activate signaling pathways such as PI3K/Akt and MAPK/ERK, promoting cell proliferation [39, 40]. Collectively, these findings confirm that the GC-HA@ZIF-8@Cur hydrogel possesses outstanding biocompatibility, establishing a solid foundation for subsequent *in vivo* experiments.

Cell migration potential was evaluated through transwell and scratch assays. Transwell experiments with L929 fibroblasts revealed increased crystal violet-positive cells in GC, HA@ZIF-8@Cur, and GC-HA@ZIF-8@Cur groups versus control, with GC-HA@ZIF-8@Cur showing maximal migration (Supplementary Figure S10). Complementary scratch assays using HUVECs demonstrated enhanced scratch closure ratios in all treatment groups, particularly GC-HA@ZIF-8@Cur, which achieved near-complete (92.824% ± 0.170%) scratch closure by 48 h (Figure 5D and E), confirming its superior migratory enhancement.

The promotion of angiogenesis plays a crucial role in enhancing diabetic wound repair processes [41]. We assessed the pro-angiogenic potential of the hydrogel materials through *in vitro* vascular network formation assays using HUVECs. The results showed that compared to other groups, the GC-HA@ZIF-8@Cur group significantly enhanced the formation of vascular structures, establishing a more complete tubular network (Figure 5F and G). Overall, these results indicate that GC-HA@ZIF-8@Cur significantly promotes cell migration and angiogenesis *in vitro*, which may be attributed to the synergistic effects of Zn^2+^ and Cur within the GC-HA@ZIF-8@Cur system.

### *In vitro* cell ROS clearance and anti-inflammatory activity analysis of hydrogels

**Figure 6.**
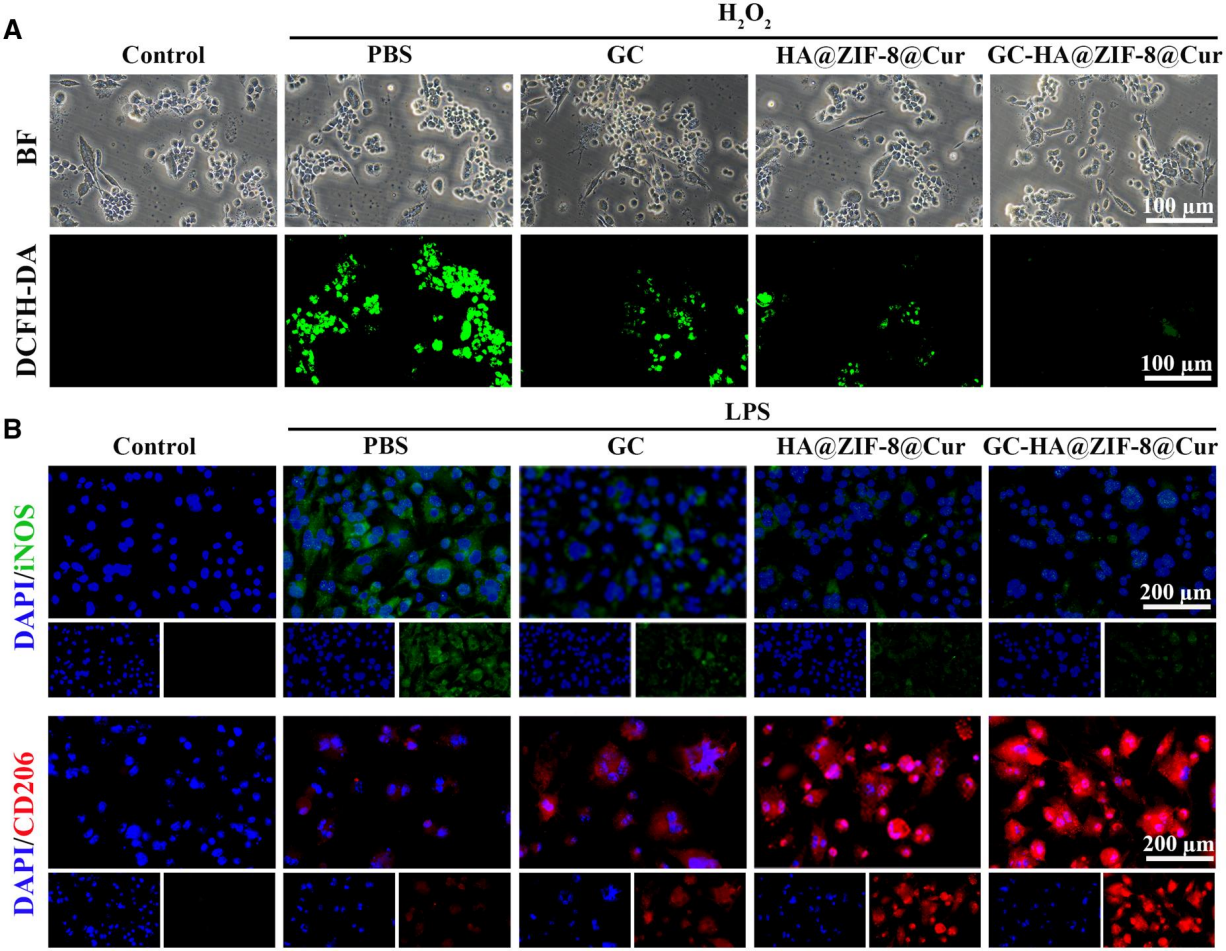
Hydrogel-mediated oxidative stress reduction and macrophage polarization modulation. (**A**) Fluorescent detection of intracellular ROS in material-treated RAW264.7 cells. (**B**) Immunofluorescence characterization of macrophage phenotypes, with iNOS indicating pro-inflammatory M1 subtype and CD206 marking anti-inflammatory M2 subtype in treated RAW264.7 cells.

The impaired healing of diabetic wounds is largely attributed to oxidative stress caused by excessive ROS accumulation. To assess the antioxidant potential of the hydrogels, DCFH-DA fluorescence staining was performed on RAW264.7 macrophages. Experimental data revealed a marked reduction in intracellular ROS levels following material treatment compared to untreated controls, demonstrating their effective ROS-scavenging capability. Notably, the ROS level in the GC-HA@ZIF-8@Cur group was markedly reduced to nearly the level of normal cells, demonstrating its excellent ROS scavenging performance (Figure 6A).

Diabetic wound healing is significantly delayed due to defective macrophage polarization from pro-inflammatory M1 to anti-inflammatory M2 phenotypes [42, 43]. We assessed the hydrogel’s immunomodulatory effects through immunofluorescence staining of RAW264.7 cells, employing iNOS (M1 marker) and CD206 (M2 marker) as phenotypic indicators [44]. The results show that material treatment substantially reduced iNOS expression while elevating CD206 levels, confirming successful M2 polarization. iNOS levels in GC-HA@ZIF-8@Cur-treated cells showed no significant difference from controls, and CD206 expression was markedly enhanced (Figure 6B), underscoring the material’s potent anti-inflammatory properties. These results collectively demonstrate that GC-HA@ZIF-8@Cur promotes wound healing through dual mechanisms: ROS scavenging and preferential induction of M2 macrophage differentiation.

### *In vivo* evaluation of the efficacy of hydrogels for diabetic wound healing

**Figure 7.**
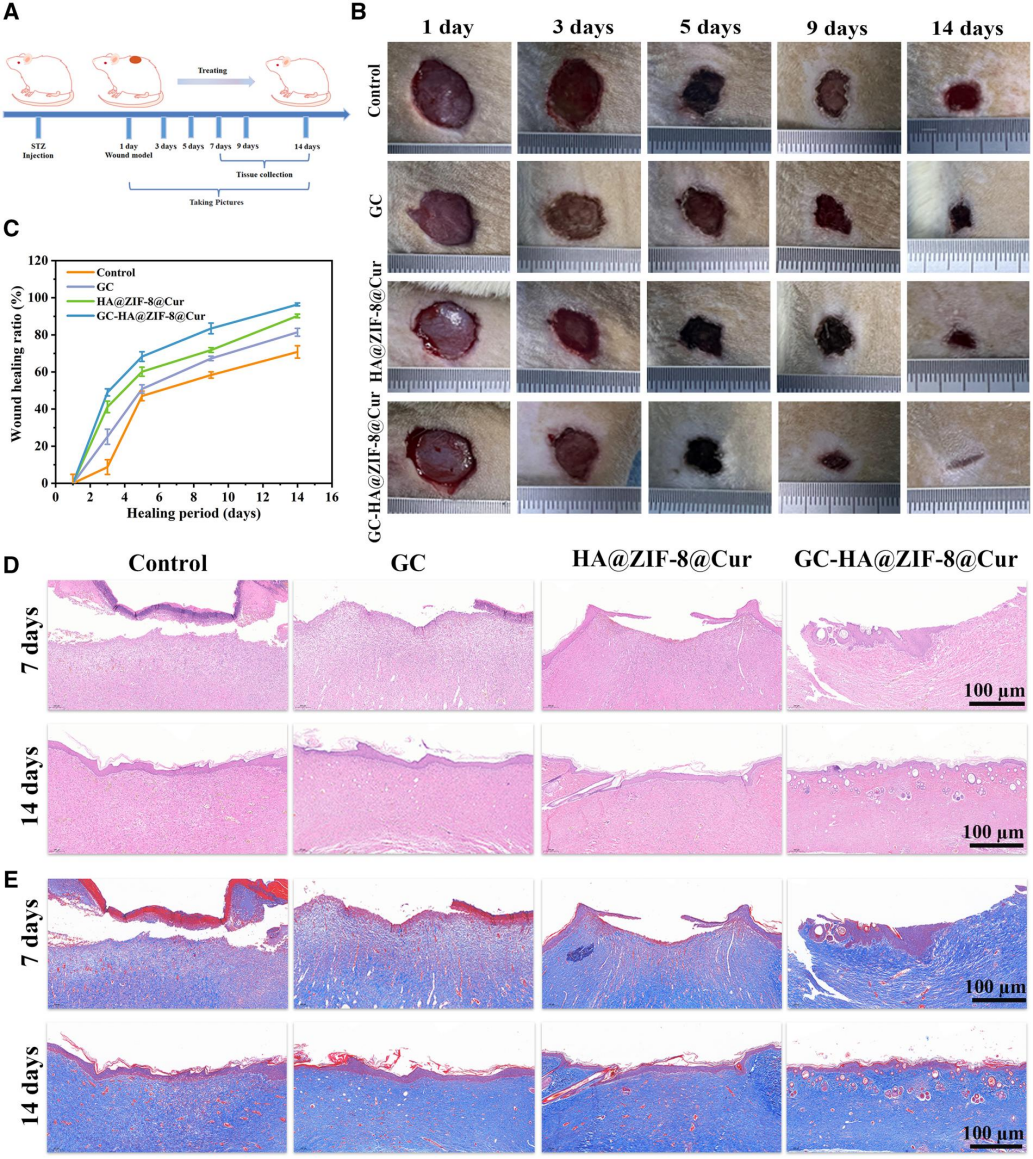
Evaluation of hydrogel-mediated wound repair in diabetic rat models. (**A**) Experimental design schematic for hydrogel treatment of diabetic wounds. (**B**) Macroscopic wound appearance following treatment at 1, 3, 5, 9, and 14 days (PBS-treated controls shown for comparison). (**C**) Quantitative assessment of wound contraction across treatment groups. (**D**) Histopathological examination via H&E staining at 7 and 14 days. (**E**) Collagen deposition analysis using Masson’s trichrome staining at identical intervals.

The wound healing performance of GC-HA@ZIF-8@Cur hydrogel was systematically examined in a diabetic rat model (Figure 7A). Three treatment groups (GC, HA@ZIF-8@Cur, and GC-HA@ZIF-8@Cur) were compared against PBS controls. Time-course analysis showed continuous wound size reduction in all groups (Figure 7B), with GC-HA@ZIF-8@Cur showing the most rapid healing by 9 days. At the study endpoint (14 days), wound closure rates reached 81.436 ± 2.108% for GC and 90.212 ± 1.010% for HA@ZIF-8@Cur, both significantly improved versus controls. The GC-HA@ZIF-8@Cur group exhibited exceptional healing efficacy, attaining 96.372 ± 0.779% wound closure—nearly complete regeneration (Figure 7B and C).

Histopathological analysis was conducted to assess wound regeneration. H&E staining revealed time-dependent healing across all groups. At day 7, enhanced re-epithelialization was observed in GC, HA@ZIF-8@Cur, and GC-HA@ZIF-8@Cur treated wounds compared to controls, with maximal epithelial regeneration occurring in the GC-HA@ZIF-8@Cur group. In contrast, GC and HA@ZIF-8@Cur groups displayed uneven wound surfaces with partial healing. By day 14, GC-HA@ZIF-8@Cur-treated wounds demonstrated complete re-epithelialization with well-developed adnexal structures including hair follicles and glandular cavities (Figure 7D). This finding indicated that GC-HA@ZIF-8@Cur had the potential to significantly facilitate wound re-epithelialization to enhance the wound healing process. In addition, Masson staining revealed that on the 7th day, the GC-HA@ZIF-8@Cur group exhibited denser and more uniformly distributed collagen deposits compared to the control group, which only demonstrated sparse collagen deposition. The GC and HA@ZIF-8@Cur groups showed collagen accumulation predominantly in the marginal regions. After 14 days of treatment, collagen deposition increased across all groups. Notably, the GC-HA@ZIF-8@Cur group displayed not only denser collagen deposition but also a more orderly arrangement compared to other groups (Figure 7E). These findings indicated that GC-HA@ZIF-8@Cur facilitated diabetic wound healing by enhancing epidermal remodeling and promoting collagen deposition.

### *In vivo* assessment of hydrogel-mediated inflammation modulation and vascularization potential

**Figure 8.**
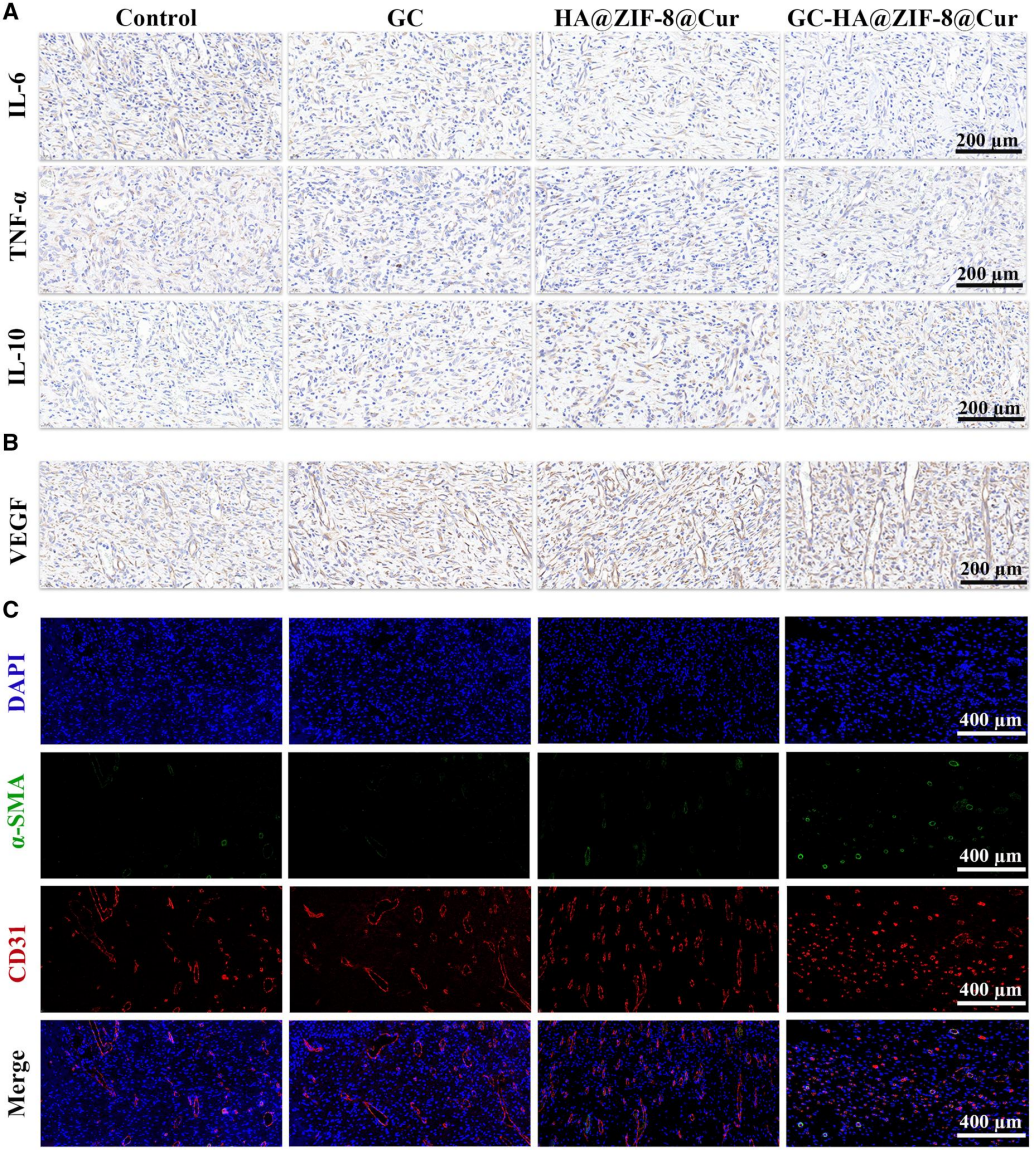
Assessment of anti-inflammatory and pro-angiogenic properties in diabetic rat models. (**A**) Day 7 immunohistochemical analysis of inflammatory cytokines (IL-6, TNF-α) and anti-inflammatory IL-10. (**B**) Immunohistochemical staining of VEGF at 7 days. (**C**) Immunofluorescence staining photographs of CD31 and α-SAM at 7 days.

The transition from inflammatory to proliferative phases plays a pivotal role in diabetic wound repair. To evaluate the hydrogels’ anti-inflammatory effects *in vivo*, wound tissues after 7 days of treatment were subjected to immunohistochemical analysis of cytokine expression (pro-inflammatory: IL-6, TNF-α; anti-inflammatory: IL-10). Quantitative results demonstrated that HA@ZIF-8@Cur and GC-HA@ZIF-8@Cur treatments significantly downregulated IL-1β and IL-6 while upregulating IL-10 expression compared to controls (Figure 8A), confirming GC-HA@ZIF-8@Cur’s superior capacity to mitigate diabetic wound inflammation.

Vascular regeneration is essential for successful wound repair [41]. To investigate the hydrogels’ angiogenic effects *in vivo*, we performed VEGF immunohistochemical analysis [35]. After 7 days, both HA@ZIF-8@Cur and GC-HA@ZIF-8@Cur treatments significantly elevated VEGF expression in wound tissues compared to controls, suggesting enhanced angiogenic activity (Figure 8B). Complementary immunofluorescence staining of endothelial marker CD31 (red) and pericyte marker α-SMA (green) [34] revealed greater vascular density in GC-HA@ZIF-8@Cur-treated wounds (Figure 8C). These results demonstrate that Cur exerts potent anti-inflammatory and antioxidant effects to eliminate vascular generation disorders, optimize the microenvironment, and thereby synergistically Zn^2+^ regulates VEGF expression, effectively promoting functional angiogenesis. Furthermore, H&E staining of major organs (lung, liver, spleen, kidney, and heart) after 14 days of GC-HA@ZIF-8@Cur hydrogel treatment showed no signs of tissue damage or abnormal alterations, further confirming the excellent biocompatibility of the hydrogel (Supplementary Figure S11).

## Conclusion

In conclusion, an intelligent composite hydrogel (GC-HA@ZIF-8@Cur) was successfully developed for diabetic chronic wound repair. By ingeniously leveraging the ROS sensitivity of GC and adjusting the hydrophilicity of the gel network, the release of HA@ZIF-8@Cur nanoparticles was accelerated. Simultaneously, by integrating the pH responsiveness of ZIF-8, the GC-HA@ZIF-8@Cur hydrogel achieved dual-responsive controlled release of Cur under diabetic wound conditions (60% cumulative release over 80 h). Moreover, the hydrogel not only exhibits excellent mechanical properties and biocompatibility but also effectively co-regulates the microenvironment of chronic diabetic wounds by scavenging ROS and promoting macrophage M2 polarization (reduced secretion of IL-6 and TNF-α). Cell culture experiments demonstrated the hydrogel’s significant enhancement of cellular migration and vascular network formation. Subsequent *in vivo* evaluation in a diabetic wound model revealed 96.372 ± 0.779% wound area reduction following 14 days of therapeutic intervention. The GC-HA@ZIF-8@Cur composite exhibited triple therapeutic effects through anti-inflammatory action, ROS scavenging, and neovascularization promotion, collectively accelerating wound repair. These findings establish an innovative microenvironment-responsive approach for advanced diabetic wound dressing development.

## Supplementary Material

rbaf098_Supplementary_Data

## Data Availability

Data will be made available on request.
