# Supplementary material for: ROS-triggered hydrophilicity switching synergizes with pH-responsive nanocarriers for therapy of diabetic wound
Source: Regen Biomater. 2025 Oct 25;12:rbaf098. doi: 10.1093/rb/rbaf098 (PMC12639545; doi:10.1093/rb/rbaf098)
Supplement: rbaf098_Supplementary_Data [file rbaf098_supplementary_data.zip › Supplementary File.docx]

**ROS-Triggered Hydrophilicity Switching Synergizes with pH-Responsive Nanocarriers for Therapy of Diabetic Wound**

Bin Yin^1, †^, Yueying Fan^1, †^, Jinfu Li^1, †^, Cheng Li^1^, Shiqiang Jiang^1^, Xiangyang Li^1^, Chao Yan^1^, Jiaxin Jiang^2^, Peng Wang^1,^ *, Chiyu Jia^1,^ *

^1^Center of Burn & Plastic and Wound Healing Surgery, The First Affiliated Hospital of University of South China, Hengyang Medical School, University of South China, Hengyang, Hunan 421001, China

^2^Guangdong Huayi Biomedical Science and Technology Center, Guangzhou, Guangdong, 511450, China

*Correspondence address. E-mail: [nxmuwp@163.com](mailto:nxmuwp@163.com) (P. W.); [jiachiyu@qq.com](mailto:jiachiyu@qq.com) (C. J.)

^†^These authors contributed equally to this work.

**

**

**Figure S1.** Determination of the Cur standard curve using a UV spectrophotometer.

**

**

**Figure S2.** Viscous property tests of GelMA, GC, and GC-HA@ZIF-8@Cur under ultraviolet light irradiation


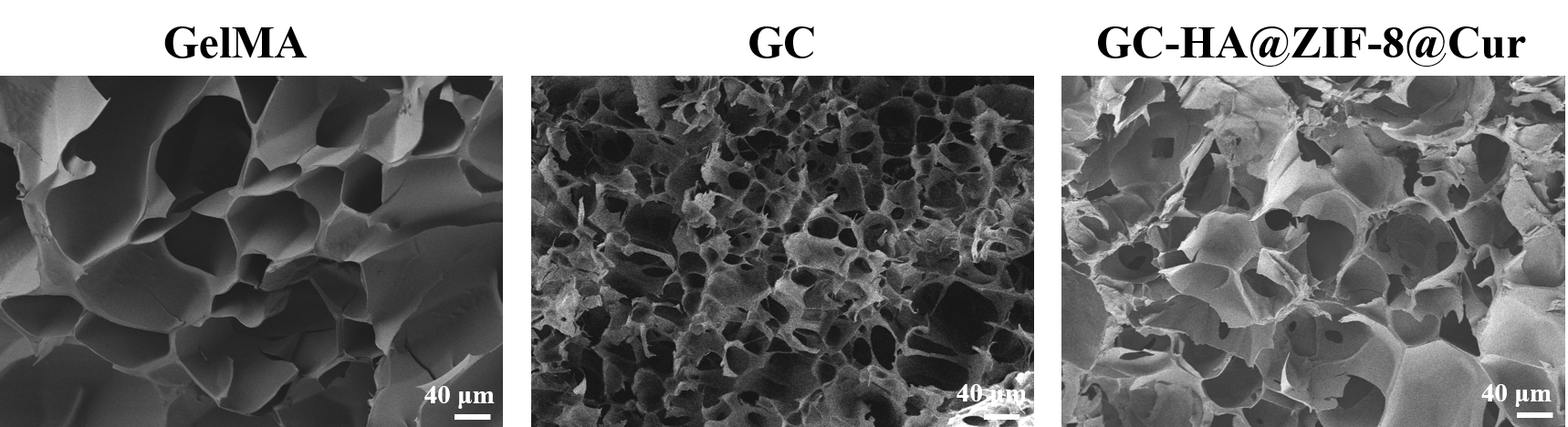


**Figure S3.** SEM images of GelMA, GC and GC-HA@ZIF-8@Cur.

**

**

**Figure S4.** Compression stress-strain curves of hydrogels with different CSLA contents


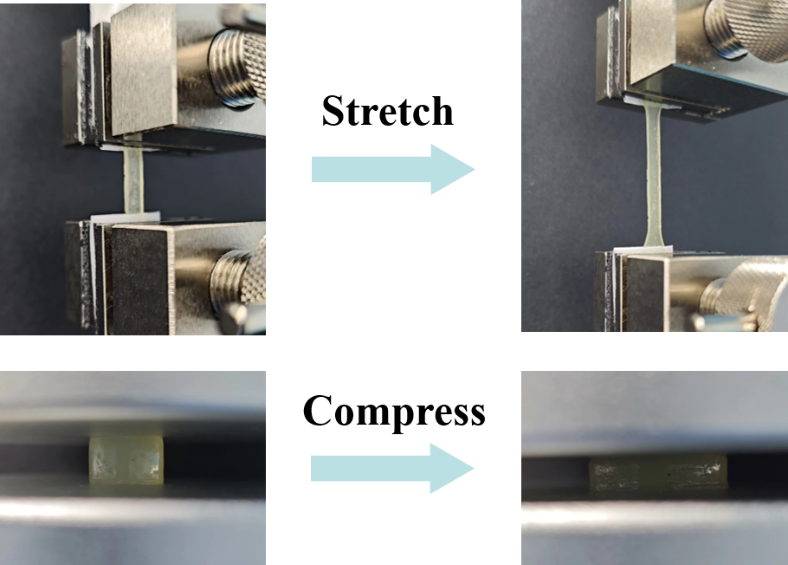


**Figure S5.** Stretch and Compression photographs of hydrogels.


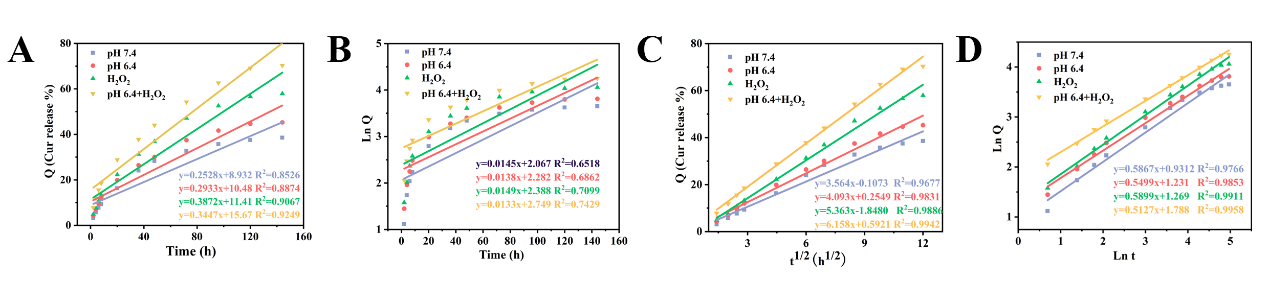


**Figure S6.** (A) Zero-order simulation of drug release in different environments of GC-HA@ZIF-8@Cur. (B) First-order simulation of drug release in different environments of GC-HA@ZIF-8@Cur. (C) Higuchi model simulation of drug release in different environments of GC-HA@ZIF-8@Cur. (D) Korsmeyer-Peppas model simulation of drug release in different environments of GC-HA@ZIF-8@Cur.


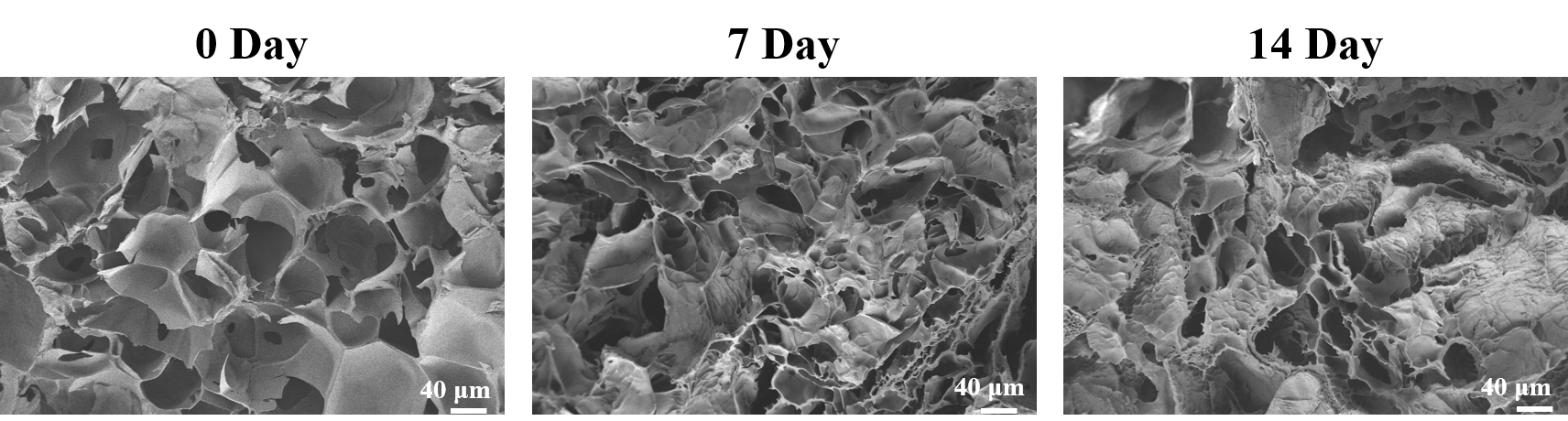


**Figure S7.** SEM images of GC-HA@ZIF-8@Cur after degradation for 0, 7, and 14 days under 37℃ PBS conditions.





**Figure S8.** The remained mass ratio of GC-HA@ZIF-8@Cur after degradation in PBS at 37 ℃ for different days.





**Figure S9.** Hemolysis ratio of GC, ZIF-8, Cur, HA@ZIF-8@Cur and GC-HA@ZIF-8@Cur.


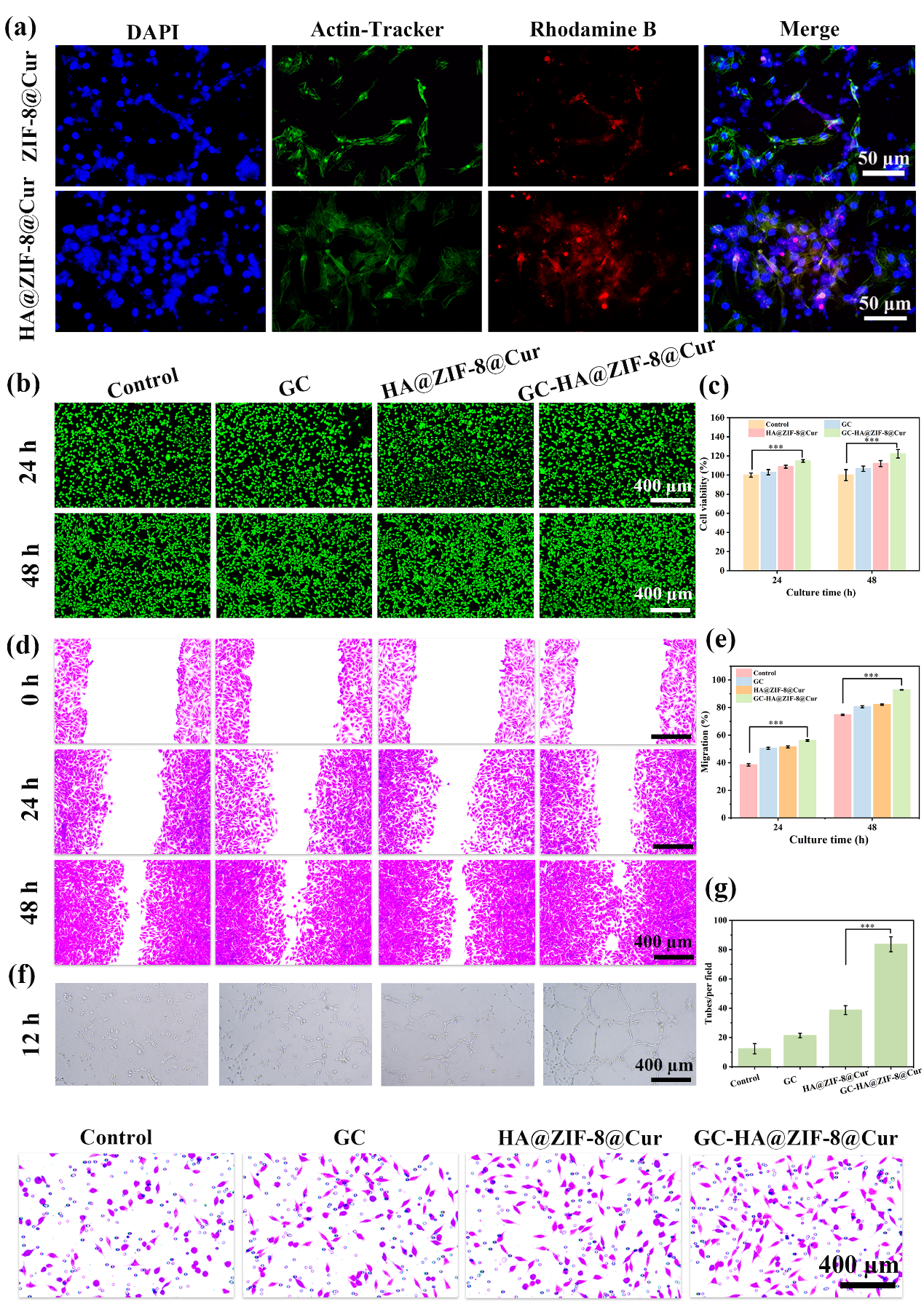


**Figure S10.** Crystal violet staining images of L929 cells treated with different materials for 24 h.


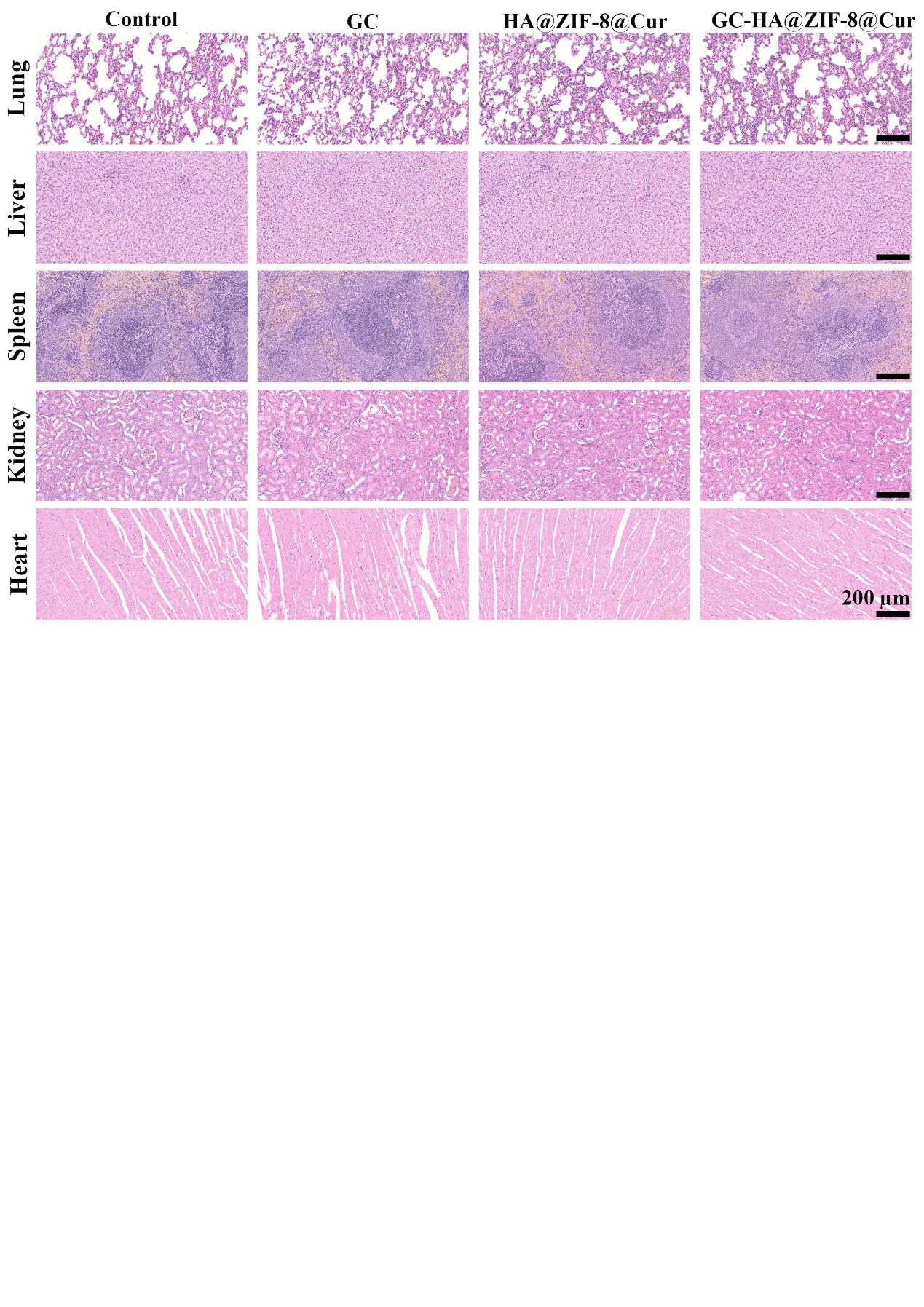


**Figure S11**. H&E staining pictures of the major organs (lung, liver, spleen, kidney, and heart) of diabetic wound rat after 14 days of GC-HA@ZIF-8@Cur hydrogel treatment.
