# Supplementary material for: ROS-triggered hydrophilicity switching synergizes with pH-responsive nanocarriers for therapy of diabetic wound
Source: Regen Biomater. 2025 Oct 25;12:rbaf098. doi: 10.1093/rb/rbaf098 (PMC12639545; doi:10.1093/rb/rbaf098)
Supplement: rbaf098_Supplementary_Data [file rbaf098_supplementary_data.zip › Response to Editors and Reviewers.docx]

Dear Editors and Reviewers,

On behalf of my co-authors, I wish to extend our heartfelt gratitude for the opportunity to revise our manuscript entitled ‘ROS-Triggered Hydrophilicity Switching Synergizes with pH-Responsive Nanocarriers for Therapy of Diabetic Wound’ (Manuscript ID: RB-2025-283). We are profoundly thankful to the editors and reviewers for their invaluable insights and recommendations on our manuscript. Taking into account the insightful remarks from the editors and reviewers, we have meticulously addressed the concerns raised and revised our manuscript accordingly. In the revised manuscript, the amended sections have been highlighted for ease of reference. Below are our responses to the comments from Editors and Reviewers. The comments are presented in black font, and our replies are articulated in blue font.

**Reviewer 1:** Comments to the Author

This study developed a pH/ROS dual-responsive intelligent hydrogel system for diabetic chronic wound therapy. This smart drug delivery strategy, which dynamically responds to pathological wound conditions, achieves synergistic effects combining anti-inflammatory, antioxidant, and pro-angiogenic properties, providing an innovative solution for establishing a stable microenvironment conducive to tissue regeneration. The system demonstrates potential for clinical applications in diabetic chronic wound therapy.

I think the results in this paper is interested to the readers.

I have some questions as follows:

1. Line 169-170: “The composite hydrogel was prepared by homogenizing GelMA (15 wt%), CSLA170 (2 wt%), and HA@ZIF-8@Cur (0.2 wt%),” Why are you utilizing this component? Have you conducted any experiments related to different components?

Thank you for your valuable comments. The composition of our composite hydrogel was determined through a combination of literature precedents and systematic optimization. The concentration of GelMA was selected based on established studies (*Biomater Res*.2023, 27, 11; Adv. *Healthcare Mater*. 2025, 14, 2402138), which demonstrated that 15 wt% GelMA provides optimal mechanical support while maintaining biocompatibility for similar applications. Chitosan has attracted significant attention due to its excellent biocompatibility, ability to promote cell proliferation and migration, and antibacterial properties (*Adv. Healthcare Mater.* 2024, 13, 2401490). After modification with lipoic acid (LA), the material not only demonstrates significantly enhanced antioxidant performance, but the disulfide bonds in LA form thioether linkages with the double bonds of GelMA, thereby endowing the GC hydrogel with ROS-responsive characteristics. Through mechanical property testing, we identified CLSA (2 wt%) as the optimal composition (Figure S4). Building upon this, we ultimately selected 0.2 wt% HA@ZIF-8@Cur as the optimal concentration to balance drug loading efficiency with biocompatibility (*Nano Today*. 2024, 56, 102235). Relevant explanatory notes have been added to the revised manuscript to improve its completeness and readability (Line 392-394).

2. Line 255：“2.16 In Vivo diabetic wound healing of the hydrogels” “To establish a diabetic wound model, male SD rats were anesthetized…” How a diabetic wound model was made? How many rats were used in each of the four experimental groups?

Thank you for your extremely detailed observation. It is of great help in improving the quality of the manuscript. To induce diabetes in the experimental rats, 32 male Sprague-Dawley (SD) rats received an intraperitoneal injection of streptozotocin (STZ, 65 mg/kg) five days prior to wound induction. Diabetic status was confirmed by sustained hyperglycemia (>300 mg/dL) through repeated blood glucose measurements. Subsequently, the rats were anesthetized using sodium pentobarbital (50 mg/kg), followed by dorsal hair removal and the creation of 15-mm full-thickness circular excisional wounds. Each of the four experimental groups used 8 rats. The relevant descriptions have been fully elaborated in the manuscript (Line 299-307).

3. Line 303-304：“while the pore size distributions of ZIF- 8@Cur and HA@ZIF-8@Cur are successively reduced”. Please give the precise data.

Thank you for your comment. We are very sorry that our description was inappropriate and might have caused misunderstandings for the readers. To better observe the pore size distribution curve, a local enlargement was performed (Figure 1g). It can be seen that the pore size distributions of ZIF-8, ZIF-8@Cur, and HA@ZIF-8@Cur are all concentrated 1.80-6.60 nm. Meanwhile, the porosity volume density of ZIF-8@Cur and HA@ZIF-8@Cur decreases successively, being 0.015 and 0.0064 cm^3^/g·nm respectively, indicating that the total pore volume of the materials changes with the loading of Cur and HA. The relevant description has been corrected and improved in the manuscript. (Line 349-353) Thank you again for your meticulous observation.

4. Line 413-414: “As shown in Figure 3f and 4g, …HA@ZIF-8@Cur has a stronger antibacterial effect than GC-HA@ZIF-8@Cur.” The difference between the two materials is GC, not Cur, and it was reported that chitosan has antibacterial effect. Why “HA@ZIF-8@Cur has a stronger antibacterial effect than GC-HA@ZIF-8@Cur”?

Thank you for your comment. Chitosan exhibits moderate antibacterial properties due to its positively charged amino groups, though its efficacy is limited by factors such as molecular weight and degree of deacetylation. In contrast, curcumin (Cur)—a natural extract from Curcuma longa—demonstrates potent antibacterial activity. Experimental results confirmed that both free Cur and HA@ZIF-8@Cur rapidly achieved significant antibacterial effects, while the GC-HA@ZIF-8@Cur system enabled sustained curcumin release, thereby providing long-term antimicrobial performance. The relevant descriptions have been improved in the manuscript (Line 484-490).

5. Line 417：“(Figure 3h and 4i).” There is no Figure 4i.

Thank you very much for your kind reminder. We feel sorry for our carelessness. In the manuscript, "Figure 3h and 4i" has been changed to "Figure 3h and 3i". (Line 492)

6. Line 472-473: “(a) Intracellular nanoparticle uptake visualized by confocal microscopy in HUVECs treated with ZIF-8@Cur and HA@ZIF-8@Cur.” Lina 439-440: “This phenomenon might be attributed to the excellent biocompatibility of HA”.
Why is there no result of GC-HA@ZIF-8@Cur? It was reported that gelatin and chitosan have also excellent biocompatibility.

Thank you for raising this important point. As a GC-HA@ZIF-8@Cur hydrogel dressing for wound healing promotion, its therapeutic efficacy can be significantly enhanced through cellular internalization of the released nanodrugs from the hydrogel network. This process would effectively amplify both anti-inflammatory and antioxidant effects, thereby accelerating wound repair. Therefore, cellular uptake experiments were conducted to evaluate the internalization efficiency of ZIF-8@Cur and HA@ZIF-8@Cur nanoparticles (Figure 4a). The results demonstrated enhanced fluorescence intensity in the HA@ZIF-8@Cur group, indicating improved cellular uptake. Furthermore, comprehensive biocompatibility assessments including hemolysis assays, live/dead cell staining, and CCK-8 assays were performed to rigorously evaluate GC hydrogel, HA@ZIF-8@Cur nanoparticles, and GC-HA@ZIF-8@Cur composite. The findings revealed that GC-HA@ZIF-8@Cur exhibited optimal biocompatibility and promoted favorable cell proliferation. These assays provide a comprehensive evaluation of material safety and cellular response (Line 504-528).

7. Line 561：“wounds (Figure 11b).”There is no Figure 11b.

Thank you for your meticulous observation, we feel sorry for our carelessness. In the manuscript, "Figure 11b" has been changed to "Figure 7c" (Line 636) and we have carefully reviewed the entire text to avoid such mistakes occurring again.

8. Line 577：in 4. Conclusion: “(60% cumulative release over 80 hours).” The conclusion should be supported by the result, I cannot find the statement in 3.2.

Thank you for pointing out this issue. The relevant result expressions have been added in 3.2 (Lines 429-430). Thank you again for your meticulous observation.

9. The first appearance of an abbreviation should be followed by its full form in parentheses，for example: MOFs, ROS, UV, LB, HUVEC etc.

Thank you for highlighting this important formatting requirement. We sincerely apologize for the oversight in not consistently defining abbreviations upon their first appearance in the manuscript. We have reviewed and revised the entire text. (Line 17, 23, 73, 104, 116 etc.) We greatly appreciate your meticulous review, which has significantly improved the clarity and professionalism of our work.

10. Have you conducted any research on the degradation rate and degradation products of this material?

We sincerely appreciate your insightful question regarding the degradation profile of our material. We have systematically investigated both the degradation ratio and degradation products of GC-HA@ZIF-8@Cur. The degradation studies demonstrated that the GC-HA@ZIF-8@Cur hydrogel gradually lost its well-ordered structure (Figure S7) when incubated in PBS at 37 °C over time, while maintaining a mass retention rate of 63.78±3.13% after 14 days (Figure S8). GC-HA@ZIF-8@Cur is primarily composed of gelatin and chitosan. Previous studies have reported that the main degradation products of gelatin in wounds are N-acetylated proline-glycine-proline (Ac-PGP) tripeptide and type-I procollagen C-propeptide (*Science and Technology of Advanced Materials*. 2023; 24(1): 2277675), while chitosan primarily degrades into glucosamine (GlcN) and N-acetyl-d-glucosamine (GlcNAc) monomers and oligomers (*Carbohydr Res*. 2020; 493: 108046). Additionally, the Zn^2+^ release ratio of 23.77±1.55% concentration in the degradation medium of GC-HA@ZIF-8@Cur was quantitatively analyzed using ICP spectroscopy at day 14 of degradation. The corresponding experimental results and analysis have been incorporated into the revised manuscript (Line 221-225, 448-457).

**Reviewer 2:**

This study proposes an intelligent nano-hydrogel system for the treatment of chronic diabetic wounds, which is based on ROS-triggered hydrophilic switching and pH-responsive drug release. The design concept of the paper is relatively novel, integrating the multifunctionality of curcumin and ZIF-8 to achieve synergistic therapy by modulating the inflammatory response and promoting angiogenesis. The overall structure of the manuscript is rigorous, the experimental methods are comprehensive, and both in vitro and in vivo validations are adequately conducted, indicating a certain level of application potential. However, there are still some issues that need to be addressed. It is recommended that the manuscript undergo detailed revisions before acceptance. My specific suggestions are as follows:

1. In Figure 4f, the contrast between the cells and the background in the tube formation assay is too low, making it difficult to distinguish the cell morphology

We sincerely appreciate your insightful comment regarding the image quality in Figure 4f. To address this concern, we have carefully repeated the tube formation assay and optimized the imaging conditions to enhance the contrast between the cells and the background. The new pictures have been replaced in the revised manuscript. We believe these modifications have resolved the issue and thank your valuable suggestion, which has strengthened the clarity of our data.

2. In the DPPH assay, what is the positive control used? Is it vitamin C? Please specify clearly.

Thank you for bringing this issue to our attention. We are very sorry for our oversight which led to the omission of important details. In the DPPH experiment, vitamin C was used as the positive control group. The relevant sections have been fully described in the manuscript (Lines 234-237).

3. Although the authors describe that Cur was loaded into ZIF-8, specific data on encapsulation efficiency (EE) and drug loading capacity (DLC) are not provided. These are fundamental parameters for evaluating a drug delivery system and should be included.

Thank you very much for your constructive comments on our work, which are very helpful for improving the quality of our manuscript. The encapsulation efficiency (EE) and drug loading capacity (DLC) of Cur loaded into ZIF-8 were 12.32 ± 0.35% and 104.08 ± 3.82 mg/g, respectively. The relevant descriptions have been refined in the manuscript (Line 159-175, 328-331).

4. Please specify the exact number of animals (n) used in each group for the in vivo experiments.

Thank you for your extremely detailed observation. It is of great help in improving the quality of the manuscript. 32 male Sprague-Dawley rats were divided into the GC, HA@ZIF-8@Cur, and GC-HA@ZIF-8@Cur groups, with 8 rats in each group. The relevant descriptions have been fully elaborated in the manuscript. (Line 299-307)

5. It is recommended that the authors include an evaluation of potential visceral toxicity.

We sincerely thank you for raising this critical point regarding systemic safety assessment. As suggested, we have now conducted a comprehensive visceral toxicity evaluation by performing histopathological analysis (H&E staining) of major organs (lung, liver, spleen, kidney, and heart) in animals treated with GC-HA@ZIF-8@Cur hydrogel (Figure S11). The results consistently demonstrated no signs of pathological abnormalities or tissue damage in all examined organs after 14 days of treatment (Line 639-642).

6. The manuscript lacks rheological characterization of the hydrogel, as well as images showing the microstructure of the freeze-dried hydrogel. It is suggested to supplement these characterizations.

We sincerely appreciate the reviewer's valuable suggestion regarding the characterization of the hydrogel. As recommended, we have now supplemented the rheological analysis of the curing process under UV light (Figure S2) and added SEM images of the freeze-dried hydrogels (Figure S3). Additionally, detailed discussions of the revised manuscript have been incorporated (Line200-202, 217-220, 380-391).

**Reviewer: 3**
Comments to the Author
The manuscript presents an innovative approach to develop a dual-responsive hydrogel system for diabetic wound healing, combining ROS/pH sensitivity with hydrophilicity switching. The study demonstrates promising in vitro and in vivo results, but several critical aspects require refinement.

1. Compare the dual-responsive release kinetics of this system with analogous hydrogels in literature (e.g., Refs. 26–27) to highlight unique advantages. Elaborate on how Zn²⁺-curcumin synergy specifically modulates VEGF expression, citing mechanistic studies on Zn²⁺-mediated endothelial cell proliferation.

Thank you for your insightful comments. Compared with the single-stimulus-responsive drug-release hydrogel reported in the literature, this study innovatively developed a ROS/pH-responsive hydrogel. Its hydrophilicity can change in response to ROS stimulation, thereby accelerating the release of nanoparticles; at the same time, combined with the inherent pH-responsive property of ZIF-8, it further synergistically enhances the drug release efficiency. We have improved the discussion of this in the introduction section of the manuscript (Lines 88-92). Regarding the Zn^2+^-curcumin synergy, curcumin exerts potent anti-inflammatory and antioxidant effects to eliminate vascularization barriers, optimize the microenvironment, and thereby work synergistically with Zn^2+^ to regulate VEGF expression, effectively promoting functional angiogenesis (*J Nanobiotechnol*, 2025, 23, 42). We apologize for any misunderstanding caused by unclear expressions and have revised the relevant sections in the manuscript (Lines 27-32, 636-638). Additionally, we have improved the discussion on the mechanism of Zn^2+^-mediated endothelial cell proliferation in the manuscript (Lines 521-526). Thank you again for your valuable comments, which have greatly improved the quality of the manuscript.

2.The HA@ZIF-8@Cur synthesis lacks details on the loading efficiency of curcumin and Zn²⁺. Please provide quantitative data (e.g., loading capacity, encapsulation efficiency) to validate reproducibility.

This comment is very valuable, as well as the important guiding significance to our manuscript. The encapsulation efficiency (EE) and drug loading capacity (DLC) of Cur loaded into ZIF-8 were 12.32±0.35% and 104.08±3.82 mg/g, respectively. Concurrently, the content of Zn^2+^ in HA@ZIF-8@Cur nanoparticles was determined to be 228.29 mg/g by ICP-OES. The relevant descriptions have been refined in the manuscript (Line 159-183, 328-331). Thank you for your valuable comments.

3.Revise ambiguous phrases (e.g., "hydrophilia accelerated release" in Scheme 1) for precision.

We sincerely appreciate your valuable comment regarding the clarity of the phrase. We have thoroughly reviewed and revised similar expressions. In Scheme 1, it is described that the thioether bonds in the hydrogel network are oxidized into sulfoxides, resulting in an increase in the hydrophilicity of the hydrogel and an acceleration of the release of nanoparticles. In Scheme 1, the parts that were prone to cause misunderstandings have been revised. At the same time, we have reviewed the entire text to prevent any ambiguous expressions from reappearing. Thank you for this helpful suggestion, which strengthens the manuscript's scientific rigor.

**Review Editor**
Comments to the Author:
This manuscript is of initial interest to the Board but will require significant revisions before the potential publication. Please submit a revised version which will be reviewed again.

Please list all the authors' names in the references instead of using "et al".

Thank you for pointing out the issue. The format of the references in the manuscript has been changed and all the authors' names have been listed.

Thank you for reading the manuscript carefully and giving us the comments. We sincerely hope the revised manuscript can meet your approval.

Yours sincerely,

Jinfu Li

Center of Burn & Plastic and Wound Healing Surgery, The First Affiliated Hospital of University of South China, Hengyang Medical School, University of South China, Hengyang, Hunan, 421001, China

E-mail: 13643418986@163.com
